# The Complete Mitochondrial Genome of endemic giant tarantula, *Lyrognathus crotalus* (Araneae: Theraphosidae) and comparative analysis

**DOI:** 10.1038/s41598-019-57065-8

**Published:** 2020-01-09

**Authors:** Vikas Kumar, Kaomud Tyagi, Rajasree Chakraborty, Priya Prasad, Shantanu Kundu, Inderjeet Tyagi, Kailash Chandra

**Affiliations:** Centre for DNA Taxonomy, Molecular Systematics Division, Zoological Survey of India, Kolkata, India

**Keywords:** High-throughput screening, Molecular evolution

## Abstract

The complete mitochondrial genome of *Lyrognathus crotalus* is sequenced, annotated and compared with other spider mitogenomes. It is 13,865 bp long and featured by 22 transfer RNA genes (tRNAs), and two ribosomal RNA genes (rRNAs), 13 protein-coding genes (PCGs), and a control region (CR). Most of the PCGs used ATN start codon except *cox3*, and *nad4* with TTG. Comparative studies indicated the use of TTG, TTA, TTT, GTG, CTG, CTA as start codons by few PCGs. Most of the tRNAs were truncated and do not fold into the typical cloverleaf structure. Further, the motif (CATATA) was detected in CR of nine species including *L. crotalus*. The gene arrangement of *L. crotalus* compared with ancestral arthropod showed the transposition of five tRNAs and one tandem duplication random loss (TDRL) event. Five plesiomophic gene blocks (A-E) were identified, of which, four (A, B, D, E) retained in all taxa except family Salticidae. However, block C was retained in Mygalomorphae and two families of Araneomorphae (Hypochilidae and Pholcidae). Out of 146 derived gene boundaries in all taxa, 15 synapomorphic gene boundaries were identified. TreeREx analysis also revealed the transposition of *trnI*, which makes three derived boundaries and congruent with the result of the gene boundary mapping. Maximum likelihood and Bayesian inference showed similar topologies and congruent with morphology, and previously reported multi-gene phylogeny. However, the Gene-Order based phylogeny showed sister relationship of *L. crotalus* with two Araneomorphae family members (Hypochilidae and Pholcidae) and other Mygalomorphae species.

## Introduction

The order Araneae is classified into two infra-orders Mesothelae (primitive spiders) and Opisthothelae (modern spiders). The infra-order Opisthothelae is further classified into two suborders Mygalomorphae and Araneomorphae with 117 families. The family Theraphosidae belongs to suborder Mygalomorphae of infra-order Opisthothelae with 992 species. Out of 992 species, 52 species are known from India^1^. The members of family Theraphosidae are commonly known as tarantulas or giant spiders for their huge body size. These giant spiders play an important role in controlling the insects^2^ and also predators on vertebrates and invertebrates^3^. The venom of these tarantulas is the main source of pharmacological research^3^.

The Pet trade of tarantulas across the globe is in great demand due to their body size, attractiveness, longitivity and for economic point of view^4^. So far, 13 species in three genera (*Poecilotheria, Thrigmopoeu*s and *Lyrognathus*) from India have been reported in the pet trade^4^. The species *Lyrognathus crotalus* is endemic to India restricted to northeast region and frequently traded. The correct identification of these tarantulas is the basic need due to their economical and medicinal values and involvement in the pet trade. However, identification of species in the absence of well-preserved specimens is not possible through morphology alone. Nowadays, molecular data are widely employed for identification of the species, resolving taxonomic ambiguities, and to infer the phylogenetic relationships. The multi-gene based analysis revealed the monophyly of Mesothelae, Opisthothelae, Mygalomorphae and Araneomorphae with adequate support and recovered Hypochilidae and Filistatidae as sister groups^5,6^. However, the phylogeny using mitogenome data, including different tree building methods, and gene orders has never been attempted. The mitochondrial genome is featured by their maternal legacy, high level of evolution and low rate of intermolecular genetic recombination, which is widely used in phylogenetic studies, population genetics, and phylogeography^7^. The circular mitochondrial genome of arthropods usually 14–19 kb in size, with 37 genes (22 transfer RNA genes, two ribosomal RNA genes, 13 protein-coding genes), and a noncoding control region. Till now, 32 mitochondrial genomes of 15 spider families are accessible in the GenBank database (https://www.ncbi.nlm.nih.gov/)^8–18^. In the present study, we sequenced the complete mitochondrial genome of *L. crotalus* using Next-generation sequencing. This is the second mitochondrial genome sequenced in the family Theraphosidae. We compared this *denovo* mitochondrial genome with 16 spider species mitochondrial genomes in 15 families to observe the codon usage patterns, gene features, gene arrangements, secondary structure of tRNAs and control region (CR), and their phylogenetic relationships.

## Materials and Methods

### Sample collection, and DNA extraction

The specimen of *Lyrognathus crotalus* was collected from the Badarpur (24.85N 92.56E), Assam, Northeast India. The morphological identification of this specimen was done by published taxonomic keys^19^, and stored in absolute ethyl alcohol at −80 °C in Centre for DNA Taxonomy, Molecular Systematics Division, Zoological Survey of India, Kolkata. DNeasy DNA Extraction kit (Qiagen, Valencia, CA) was used for genomic DNA extraction and quantified by dsDNA high-sensitivity kit (Thermo Fisher Scientific, MA, USA) in Qubit fluorometer. In this study, no prior permission was required for the collection as the species is neither endangered nor protected species in IUCN Red List or Indian Wildlife Protection Act, 1972.

### Mitochondrial genome sequencing and assembly

The Genotypic Technology Pvt. Ltd. Bangalore, India (http://www.genotypic.co.in/) had carried out the sequencing. The sequencing and assembly protocol was followed by our previous study^20,21^. The whole genome library of genomic DNA was sequenced using Illumina Hiseq. 2500 (2 × 150 base paired-end reads) (Illumina, USA) platform which yielded ~14 million reads. The TruSeq DNA Library Preparation kit (https://support.illumina.com/downloads/truseq) was used for the construction of the paired-end library with standard protocols. The trimming and filtering of the raw sequencing reads were done by using the NGS-Toolkit^22^ to removing adapter contamination and low-quality reads with a cutoff of Phred quality scores of Q20. Burrows-Wheeler Alignment (BWA) tool^23^ screened the high quality reads (1.4 million) using Seqtk (https://github.com/lh3/seqtk) and down sampled high-quality reads and assembled with SPAdes 3.9.0^24^, using *Ornithoctonus huwena* mitochondrial genome (NC_005925.1) as a reference.

### Annotation, secondary structure prediction, and comparative studies

The annotation of PCGs and rRNAs were done by using MITOS web-server^25^. The annotation of the tRNA genes was the most difficult steps to define their location and gene boundaries. To search the tRNAs in the mitochondrial genome, the tRNAscan-SE 1.21^26^ was used, but incapable to detect tRNAs. Further, we have used other spider tRNA sequences from GenBank to confirm the locations and boundaries of each tRNAs. Further, the anticodon arm motifs (or sometimes only the 3-bp anticodon sequence) were examined manually which were conserved among all spiders.

The start and stop codons of PCGs were observed by using the ClustalX program^27^. The PCGs of *L. crotalus* with other spider species were aligned by MEGAX^28^. For acquiring the accession number of the complete annotated *L. crotalus* mitochondrial genome from GenBank, the Sequin submission tool (http://www.ncbi.nlm.nih.gov/Sequin/) was used. The circular image of *L. crotalus* mitochondrial genome was drawn by using online server CGView^29^ (http://stothard.afns.ualberta.ca/cgview_server/). The length and locations of spacer regions (overlapping and intergenic) of *L. crotalus* mitochondrial genome were detected manually.

The nucleotide composition, codon usages, relative synonymous codon usage (RSCU) was done by MEGAX. To calculate the skewness, we used the formula: AT skew = (A − T)/(A + T) and GC skew = (G − C)/(G + C)^30^. Codon usage bias was evaluated by calculating of effective number of codon (ENc) with the DnaSP6.0^31^. The relative synonymous codon usage (RSCU) graph is plotted in Microsoft Office Excel. The ratios of non-synonymous substitutions (Ka) and synonymous (Ks) substitutions were estimated in DnaSP6.0. The transition and transversion ratio versus genetic distance calculated by using DAMBE5^32^. We predicted the secondary structure of tRNAs and CR for *L. crotalus* mitochondrial genome. The tRNAs secondary structures were predicted by VARNA 3.93^33^. The prediction of secondary structure of CR was done by The Mfold web server^34^.

### Preparation of data sets, model selection, phylogenetic analyses

Out of 32 available spider mitogenomes in the global database, 16 species mitochondrial genomes were retrieved and used in the present dataset based on representative families^8–18^. The mitogenome of *Limulus polyphemus* (order: Xiphosura) were also retrieved from GenBank and used as an out-group^35^ (Table S1). The four data sets were prepared for phylogenetic analysis: (1) PCGs without GBlock^36^, 11453 bp; (2) PCGs without GBlock (third codon position excluded), 7640 bp; (3) PCGs with GBlock, 8706 bp; (4) PCGs with GBlock (third codon position excluded), 5804 bp. The PartitionFinder version 2.1.1^37^, with the greedy algorithm was used to find the best substitution models for Bayesian Inference (BI) and maximum likelihood (ML). The PartitionFinder analyses: codon positions for each PCGs (13 genes × 3 codons = 39 partitions), PCGs excluding 3^rd^ codon position (13 genes × 2 codons = 26 partitions) (Table S2). We used the online web portal The CIPRES Science Gateway v.3.1 (www.phylo.org/sub_sections/portal/) to perform Mr. Bayes 3.2 for BI analysis^38^ and ML analysis using IQ-tree web server using four data sets^39^. The phylogenetic tree was visualized and edited using FigTree v1. 4.2^40^ (http://tree.bio.ed.ac.uk/software/figtree/). We used the MLGO web server^41^ for gene order based phylogeny (http://www.geneorder.org/server.php) (Table S3).

### Gene arrangement analysis

Three methods were applied to see the gene arrangement scenario: (1) CREx^42^ based on the common interval; (2) mapping the gene boundaries on the gene order to see the unique, derived and ancestral gene boundaries between the ancestral arthropod *L. polyphemus* and other taxa of spiders (Tables S4 and S5); and (3) TreeREx Analysis^43^ based on common used to evaluate evolutionary pathways. TreeREx analysis is the extended version of CREx with default settings was performed: strong consistency method applied (-s); weak consistency method applied (-w); parsimonious weak consistency method applied (-W); get alternative bp scenario for prime nodes (-o); maximum number of inversions (-m = 0) + TDRL scenarios considered. Three levels of nodes were inferred, green colour nodes were consistent, yellow colour 1-consistent nodes, and the red colour fallback nodes.

## Results and Discussion

### Genome structure, organization and composition

**Figure 1:**
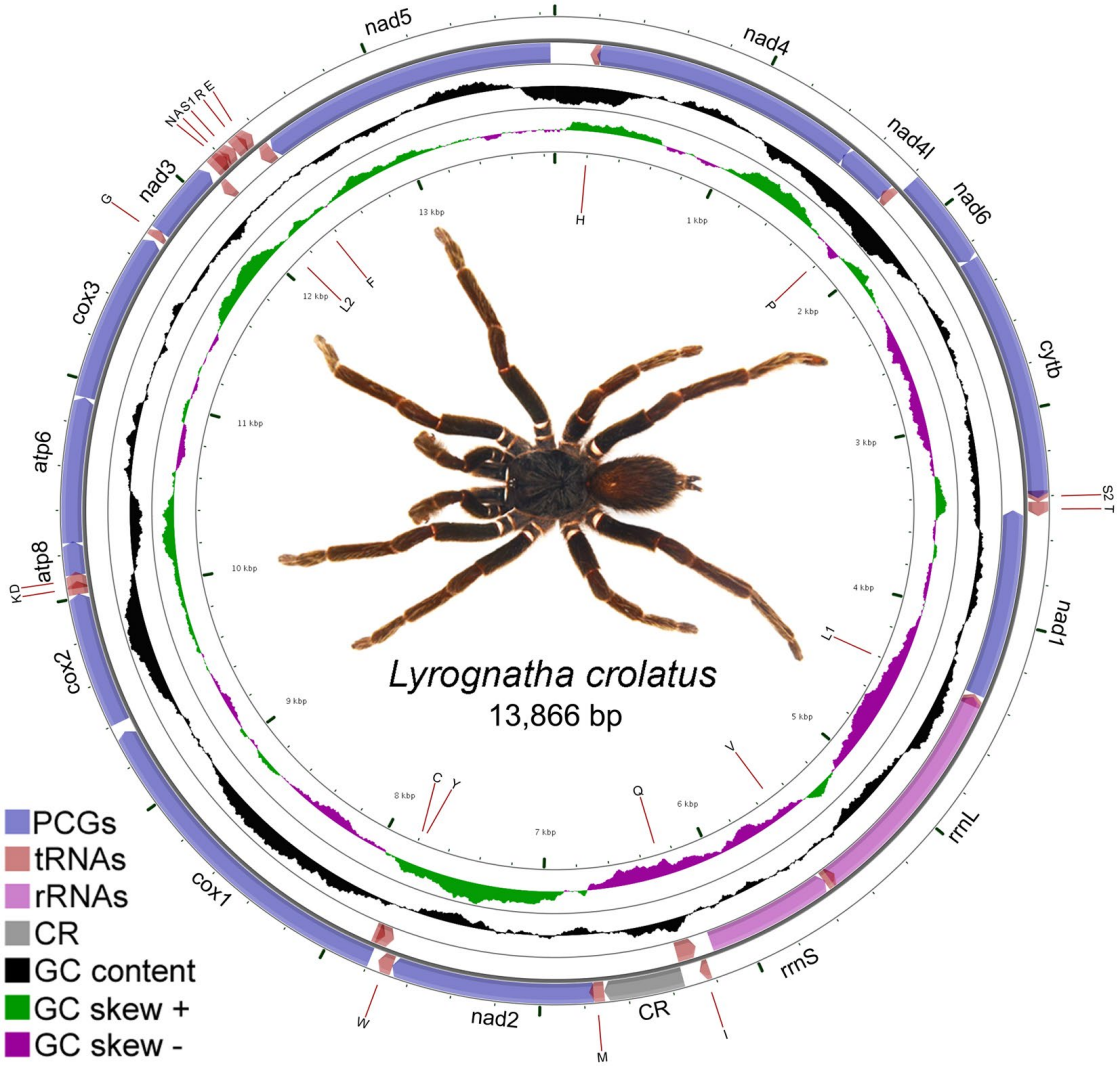
The circular representation of the complete mitochondrial genome of *L. crotalus*. Direction of gene transcription is indicated by arrows in entire complete genome. PCGs are shown as purple arrows, rRNA genes as pink arrows, tRNA genes as peach color arrows and CR regions as gray rectangles. The GC content is plotted using a black sliding window, as the deviation from the average GC content of the entire sequence. GC-skew is plotted using a colored sliding window (green and orchid color), as the deviation from the average GC skew of the entire sequence. The figure was drawn using CGView online server (http://stothard.afns.ualberta.ca/cgview_server/) with default parameters. The species photograph was taken by the fourth author using Leica Microscope EZ4 HD with Leica software application suite (LAS EZ) and edited manually in Adobe Photoshop CS 8.0.

*L. crotalus* complete mitochondrial genome (accession number MN072398) is 13,866 base pairs (bp) in length. This is the smallest mitochondrial genome of spider among all published mitochondrial genomes so far due to the presence of extremely truncated tRNAs. It included 37 genes: 13 PCGs, large and small rRNAs, 22 tRNAs and one non-coding region (D-Loop) with the origin of light-strand replication (OL) (Fig. 1, Table 1). The majority strand contains 22 genes and minority with 15 genes (Table 1). The AT and GC content of nucleotide was 68.74% and 31.26%, respectively (Table S6), like other spider mitochondrial genomes. The highest AT content observed in tRNAs (69.68%,), followed by PCGs (68.72%), rRNAs (68.66%), and CR (67.67%). The mitochondrial genome showed positive AT (0.07) and negative GC (-0.36) skewness (Table S6) in contrast to other spider mitochondrial genome.

**Table 1:** List of annotated mitochondrial genes of *L. crolatus* and its characteristic features.

Gene	Strand	Location		Size (bp)	Anti-codon	Start Codon	Stop Codon	IGN
		start	stop					
*H*	−	172	220	49	GTG			−17
*nad4*	−	204	1511	1308		TTG	TAA	−8
*nad4L*	−	1504	1773	270		ATT	NOT Found	−12
*P*	−	1762	1816	55	TGG			8
*nad6*	+	1825	2271	447		ATT	TAA	−1
*cytb*	+	2271	3410	1140		ATG	TAA	−35
*S2*	+	3376	3429	54	TGA			−3
*T*	+	3427	3492	66	TGT			−27
*nad1*	−	3466	4374	909		ATA	TAA	1
*L1*	−	4376	4432	57	TAG			−41
*rrnL*	−	4392	5519	1128				−34
*V*	−	5486	5547	62	TAC			−5
*rrnS*	−	5543	6169	627				47
*I*	+	6217	6262	46	AAT			−18
*Q*	−	6245	6343	99	TTG			0
CR		6344	6708	365				0
*M*	+	6709	6777	69	CAT			−20
*nad2*	+	6758	7690	933		ATT	TAG	−2
*W*	+	7689	7758	70	TCA			−33
*Y*	−	7726	7806	81	GTA			−35
*C*	−	7772	7824	53	GCA			−28
*cox1*	+	7797	9353	1557		ATA	TAG	48
*cox2*	+	9402	10019	618		ATG	TAG	−3
*K*	+	10017	10078	62	CTT			−33
*D*	+	10046	10121	76	GTC			−15
*atp8*	+	10107	10259	153		ATT	TAA	−7
*atp6*	+	10253	10921	669		ATG	TAG	1
*cox3*	+	10923	11708	786		TTG	TAA	11
*G*	+	11720	11764	45		GGA		−5
*nad3*	+	11760	12110	351		ATT	TAA	−22
*L2*	−	12089	12142	54	TAA			−6
*N*	+	12137	12215	79	GTT			−48
*A*	+	12168	12231	64				−23
*S1*	+	12209	12269	61	GCT			−4
*R*	+	12266	12334	69	CGA			−26
*E*	+	12309	12370	62	TTC			−39
*F*	−	12332	12400	69	GAA			−1
*nad5*	−	12400	13845	1446		ATT	TAA	

The PCGs and rRNA genes are represented by standard nomenclature, tRNAs are represented followed by the IUPAC-IUB single letter amino acid codes. (+) values in strand represent as heavy (H) and (−) values represent as light (L). IGN represents (+) values as intergenic nucleotides and (−) values as overlapping regions. CR represents the control region.

### Protein-coding genes

The total length of PCGs was 10,587 bp in *L. crotalus*. The ATN start codons were used by most of the PCGs except *cox3*, and *nad4* with TTG. Comparative study revealed that ATN and TTG start codon used by most of the PCGs of spider mitochondrial genomes. TTT start codon used by only *cox1* (*Tetragnatha maxillosa*^17^); TTA used by *cox1* (*Phyxioschema suthepium*, *Ornithoctonus huwena*^8^, *Agelena silvatica*^11^, *Carrhotus xanthogramma*^13^, *Selenops bursarius*^16^, *Oxytate striatipes*^15^); GTG used by *cox2* (*Selenops bursarius*, *Pholcus phalangioides*), *cytb* (*Ornithoctonus huwena*), *nad2* (*Pholcus phalangioides*), *nad6* (*Oxyopes sertatus*^14^), *atp6* (*Pholcus phalangioides*); CTG used by *cox1* (*Neoscona theisi*^10^*, Liphistius erawan*); CTA used by *cox1* (*Calisoga longitarsis*^8^). The stop codons TAA, TAG, and T(AA) were used commonly by most of the PCGs as observed in other spiders (Table S7).

### Codon usage bias and mutations

The use of codons or codon usage bias is a fundamental phenomenon in nature^44,45^. The main influencing forces for codon usages are the mutation pressure and natural selection. Codon usage bias can be triggered by a number of other factors such as the content of nucleotides, gene length and their function, and the external environment^45^. We investigated the GC content to study the nucleotide distribution of all three codon positions of PCGs for 17 spider mitochondrial genomes (Fig. S1). The average GC content was 29.02%, while the value of GC1s, GC2s and GC3s were 35.98%, 34.90% and 20.02% respectively. The codon frequency ending with A/T is higher than G/C due to the AT rich segments which leads to the high codon bias^46,47^. The comparative study of spider mitochondrial genomes revealed 21 codons (9 with A-ending, 12 with U-, and none with G- or C-ending) with high frequency (Table S8). This result suggested that compositional constrain may play an important role in the codon usage patterns in spider species.

**Figure 2:**
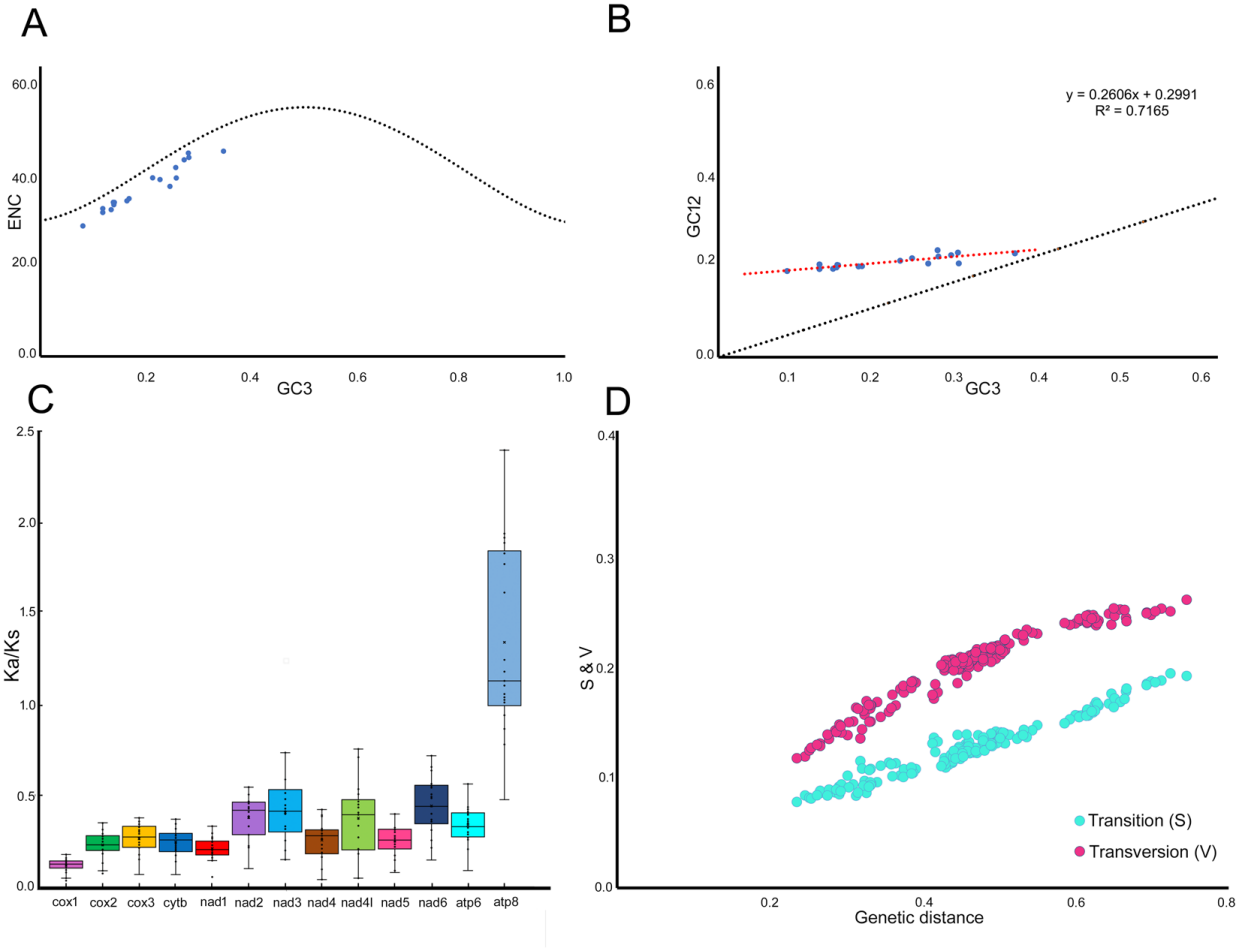
**(A)** ENC versus GC3 plotting of concatenated PCGs of spider mitochondrial genomes; **(B)** Neutrality plot of concatenated PCGs of spider mitochondrial genomes; **(C)** Box plot for pairwise divergence of Ka/Ks ratio for 13 PCGs of spider mitochondrial genomes; **(D)** Genetic distance versus transition-transversion ratio of 13 PCGs spider mitochondrial genomes. The figure was edited in Adobe Photoshop CS 8.0.

The average of the Effective Codon number (ENc) values for all the PCGs was 42.97, which indicate a strong codon bias, ranging from 39.31 to 46.83. We plotted ENc-GC3s value to explain the relationship between nucleotide composition and codon bias (Figs. 2A and S2). The result specified that not only mutation, but other factors like natural selection might be involved in shaping the codon bias in spider mitochondrial genomes. To confirm the relation between GC12 and GC3 and explain the mutation-selection equilibrium in shaping the codon usage bias, we used neutrality plot analysis (Fig. 2B). This plot indicates that the genes have a wide range of GC3 value distributions, ranging from 8.1% to 34.7%, and also showed the positive correlation between GC12 and GC3 (r = 8455, p < 0.01). Moreover, the slope of the regression line of the entire coding sequences was 0.2516. So, the natural selection may probably dominate the codon bias rather than mutation bias^48^.

### Non-synonymous and synonymous substitutions

To investigate the selective pressure and evolutionary relation of the homogenous or heterogeneous species, non-synonymous and synonymous substitutions (Ka/Ks) ratio was used^49^. Our result showed the average Ka/Ks ratio ranging from 0.122 ± 0.03 in *cox1* to 0.443 ± 0.14 in *nad6* gene and the resulted following order: *cox1* < *nad1* < *cox2* < *nad5* < *cytb* < *cox3* < *nad4* < *atp6* < *nad4l* < *nad3* < *nad2* < *nad6* < *atp8*. This result indicated that the 13 PCGs excluding *atp8* of all spider mitochondrial genomes including *L. crotalus* were evolving under purifying selection (Fig. 2C). The value of Ka/Ks for all the PCGs was below one, indicating the mutations swapped by synonymous substitutions. The *cox1* gene with low Ka/Ks ratio represent fewer changes in amino acids and hence widely used as a potential molecular marker for species identification and phylogenetic analysis^50,51^. The transversion and transition plot against the genetic distance showed a linear relationship for PCGs (Fig. 2D). The value of the substitution saturation index for the combined dataset of all PCGs of 17 spider mitochondrial genomes (Iss = 0.4261) was significantly lower than the critical values (Iss. cSym = 0.8367 or Iss.cAsym = 0.6659). Hence, the combined data is suitable for phylogenetic analysis.

### Ribosomal and transfer RNA genes

Two rRNAs were observed in *L. crotalus* and other spider mitochondrial genome. The large ribosomal *rrnL* (16S RNA) placed between *trnV* and *trnL1*, was 1128 bp long; the small *rrnS* (12S RNA) between *trnI* and *trnV*, was 627 bp long (Table 1). *L. crotalus* has 22 tRNAs (total length 1,329 bp), ranging from 41 to 99 bp in length. (Table S5).

The prediction of the secondary structure and gene boundaries for all the tRNA genes was extremely difficult, as truncated at their 3′ end and lack of proper base pairing at the 5′ end (Fig. S3). The extreme truncation or atypical structure of tRNA is very peculiar in Arachnids mitochondrial genomes, including Araneae^9,52^. Seventeen of the 22 tRNAs has atypical secondary structures, missing either a D-arm or T-arm. The majority of tRNAs also have mismatches on T-arm, D-arm, acceptor arm or anticodon arm. The lack of acceptor arm or very poorly paired arm was observed in the following genes (*trnL1*, *trnS1, trnE, trnL2, trnI*). The lack of T-arm and loop were observed in 13 genes (*trnC*, *trnD*, *trnF*, *trnG*, *trnH*, *trnK*, *trnL2*, *trnN*, *trnP, trnI, trnY, trnQ* and *trnV*), which was inferred by TV-replacement loops. The lack of DHU loop observed in *trnN* whereas, lack of DHU arm and loop observed in *trnS1*, *trnI* (Fig. S3). Out of 22 tRNAs, boundaries of nine genes were overlapped with the adjacent gene on both the ends. The gene overlaps or quantity of truncation in each tRNA gene were differ from species to species. *trnE* showed overlaps of 39 nucleotides (nt) with its neighboring gene (*trnF*) on the same stand at 5′ end and 26 nt overlap with *trnR* gene on the opposite strand at the 3′ end and. After overlapping on both the ends, only four nt was remaining which exclusively denoted for *trnE*. *trnC* showed overlaps of 28 nt at the 5′ end with its neighboring gene (*cox1*) on the opposite strand and 35 nt overlap at the 3′ end with *trnY* gene on the same strand. After overlapping on both the ends, no nucleotide was remaining for *trnC*.

### The A + T-rich region

**Figure 3:**
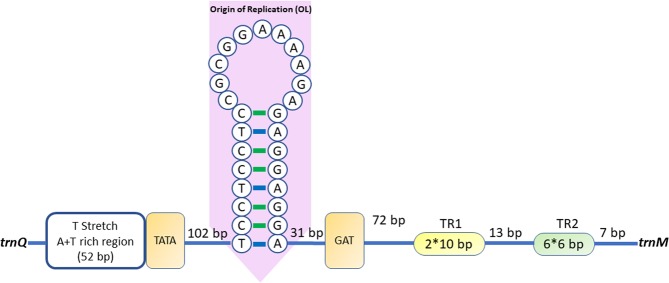
Secondary structure of the control region of *L. crotalus*. The figure was edited in Adobe Photoshop CS 8.0.

The A + T-rich region in mitochondrial genome is important for the initiation of replication in Metazoans^53,54^, and also called the control region (CR). It is located between *trnM* and *trnQ* in *L.crotalus*, spans 356 bp with 67.67% AT content and showed positive AT/GC skew (0.04/0.15), indicating an obvious bias towards the use of A and G. The non-coding region named replication origin of L-strand (OL) region was also found. The “OL” region (TCCTCCTCCGCGGAAAAGAGAGGAGGA) is 27 bp in length and has the potential to fold into a stem-loop secondary structure (Fig. 3). Apart from the conserved elements in A + T-rich region, tandem repeats were also found to be a characteristic of A + T-rich regions. In *L.crotalus*, two tandem repeats of 10 bp (ATTTTTATTC) and six tandem repeats of 6 bp (CATATA) were present at the 3′ end upstream of the *trnQ*. Further, this motif was also detected in other eight species (*A*. *angulatus*, *C. longitarsis, C. xanthogramma*, *H. thorelli*, *N. theisi*, *O. huwena, P*. *phalangioides, S*. *bursarius*). All elements which are related to the regulation of transcription and control of DNA replication in the A + T-rich region were arranged in the conserved order as compared to other Opisthothele spiders.

### Phylogenetic analyses

**Figure 4:**
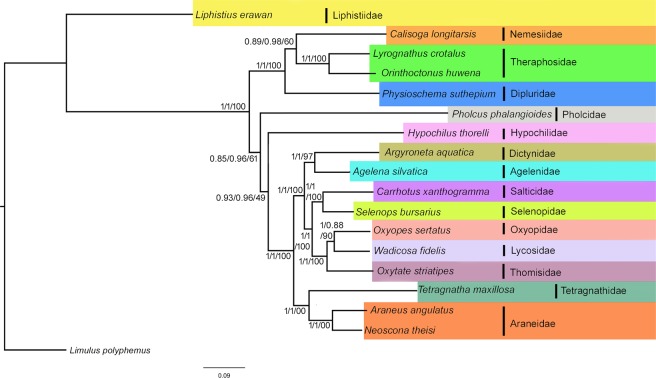
Linearized view of gene arrangements of spider species and ancestor (*Limulus polyphemus*) in correspondence with Gene order based tree MLGO phylogeny. Species with identical gene order in correspondence to MLGO phylogeny. The PCGs and rRNAs are represented by their standard nomenclature with light orange and green color respectively. The rRNAs are represented by dark grey color. The CRs are represented by pink color. Derived gene boundaries are numbered with numerals, and plesiomorphic gene block are underline and numbered with alphabet. The figure was edited in Adobe Photoshop CS 8.0.

Eight phylogenetic trees based on four datasets and two inference methods, Maximum likelihood (ML) and Bayesian Inference (BI) were generated (Figs. 4 and S4–S6). All the analyses supported the monophyly of suborders Opisthothelae with high bootstrap support and posterior probability. The infraorder Mygalomorphae was recovered as monoplyletic in all the analyses. The Araneomorphae was recovered paraphyletic in all the analyses except BI-2, BI-4, ML-4 (Fig. 4). The paraphyly in the Araneomorphae is due to the varied position of family Hypochilidae and Pholcidae. The family Hypochilidae is first to branch from the other taxa of spiders and leaving two clade with strong posterior probabilities and bootstrap support in BI-1, 3, ML-2 (Figs. S4 and S6). The first clade of Mygalomorphae + family Pholcidae of Araneomorphae and; second clade of Araneomorphae. In two analyses (ML-1, 3), the families Pholcidae and Hypochilidae were grouped with Mygalomorphae taxa with low bootstrap support (Fig. S5). In all the analyses *L*. *crotalus* is cladded with *O*. *huwena* and showed sister relationship with *C*. *longitarsis* (Nemesiidae) and *P. suthepium* (Dipluridae). The estimated tree from the mitochondrial genome is well supported by previously generated multilocus phylogeny^5^. However, the genital structure is the distinguishable feature of the Mygalomorphae and Araneomorphae spiders with three types of genitalia^5,55^: Haplogyne (simple) in Mygalomorphae, Entelegyne (two separate ducts for copulation and fertilization) and non-entelegyne (single duct for both copulation and fertilization) in Araneomorphae. The estimated trees in this study are also supported by a previous study showing the monophyly of entelygyne taxa. However, non-entelygyne Araneomorphae (Pholcidae and Hypochilidae) without cylindrical gland spigots always grouped with Haplogyne (Mygalomorphae).

**Figure 5:**
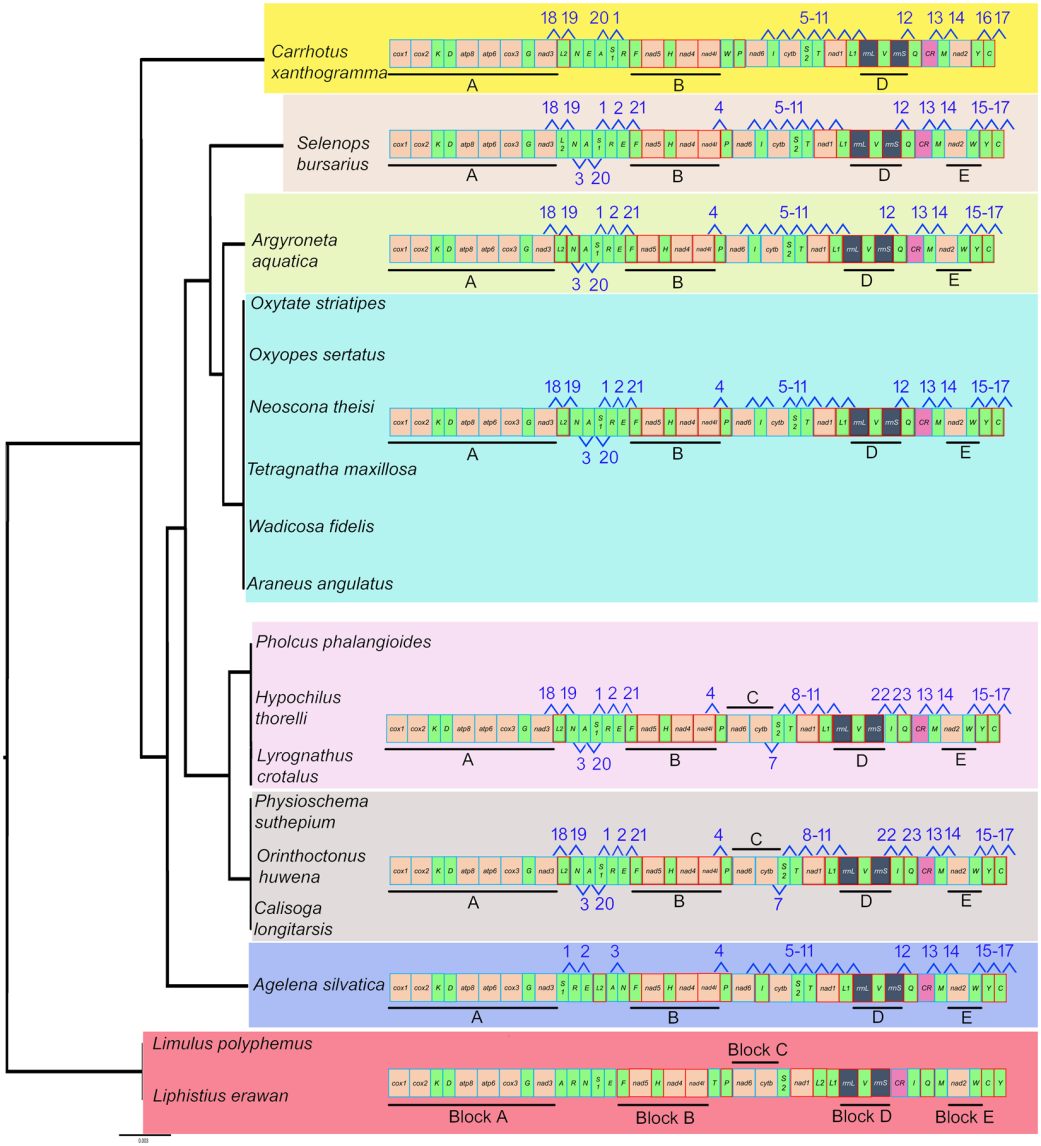
Bayesian Phylogenetic tree inferred by 13 PCGs of spider mitochondrial genome. The tree is drawn to scale with posterior probabilities and bootstrap support values indicated along with the branches in the order (BI-2/BI-4/ML-4). The figure was edited in Adobe Photoshop CS 8.0.

The gene order based MLGO phylogeny indicated the monophyly of suborder Opisthothelae (Fig. 5, Table S3). The family Salticidae and Agelenidae of Araneomorphae showed sister relationship with other spider taxa. The Further two clades were formed: 1) Mygalomorphae + Hypochilidae and Pholcidae; 2) other Araneomorphae taxa. The close relationship of Araneomorphae non-entelegyne taxa (Pholcidae and Hypochilidae) with Mygalomorphae was also indicated in the sequence based phylogenetic analyses. The studied tarantula species, *L. crotalus* revealed a close relationship with the Hypochilidae and Pholcidae in the MLGO phylogeny as they share the same gene order. MLGO phylogeny also revealed paraphyly of entelygyne taxa^5^. The BA and ML phylogenies are discordant with MLGO phylogeny and indicated the cladding of *L*. *crotalus* (Theraphosidae) with non-entelegyne taxa. However, more mitochondrial genome data of both non- entelegyne and haplogyne taxa would cover the consistent sign of deep phylogenetic relationship between them.

### Gene arrangement

Three methods were applied to see the gene arrangement (1) CREx; (2) mapping the gene boundaries on the gene order; (3) TreeREx Analysis.

### CREx

The transpositions, inversions, and inverse transpositions are the characteristic pattern of gene arrangement in the mitochondrial genome^56^. To explain the transpositions in gene arrangement, the Tandem Duplication–Random Loss (TDRL) is the most commonly known process^42^. By comparing the gene order of *L. crotalus* with the putative ancestral arthropod mitochondrial genome (*L. polyphemus*) by using CREx analysis, five tRNAs (*trnT, trnL2*, *trnI*, *trnQ*, and *trnY*) were found to transposition and one TDRL event in block *trnN*-*trnA*-*trnS1*-*trnR*. The *trnI* changed its position to a new location between *rrnS* and *trnQ* and also moved from majority to minority strand. Inverse transposition of *trnI* was also observed in *A. silvatica, C. longitarsis, O. huwena*, and *P. suthepium*. The comparative study of gene arrangement revealed that *L. crotalus* (Theraphosidae) shared the gene order with following species, *O. huwena* (Theraphosidae)*, P. suthepium* (Dipluridae)*, C. longitarsis* (Nemisiidae), *H. thorelli* (Hypochilidae) and *P. phalangioides* (Pholcidae). Further, CREx analysis revealed the inversion of *trnI* within mygalomophs, Hypochilidae and Pholcidae. Hence, the studied species *L*. *crotalus* is more inclined towards family Hypochilidae and Pholcidae (Table S4).

### Mapping of the gene boundaries

**Figure 6:**
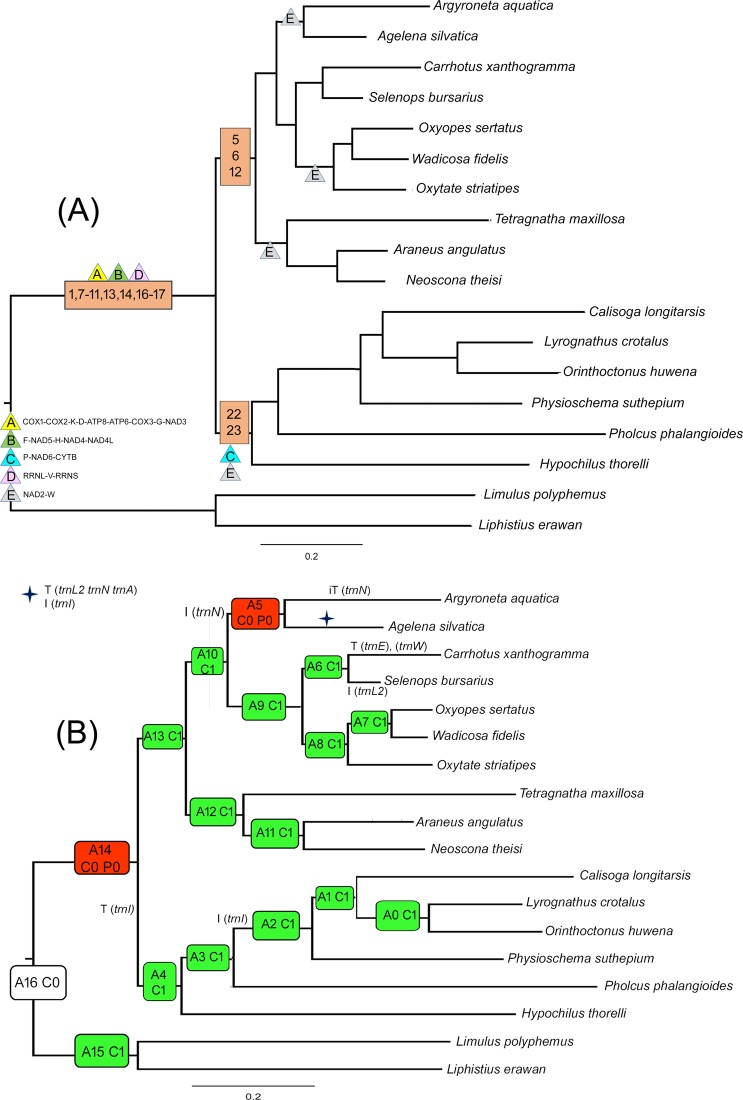
(**A**) Mapping genome rearrangements and reductions onto an evolutionary tree of the spiders. Maximum likelihood tree (ML-1) is used for representation of the plesiomorphic and synapomorphic gene blocks. Synapomorphic gene blocks are labelled with numerals, and plesiomorphic with alphabet of different colours. (**B**) Original output of TreeREx analysis of spiders gene order. Maximum likelihood tree (ML-1) is used for representation and analysis. The rearrangements on the branches are given as T for a transposition and TDRL for tandem-duplication-random-loss events; green nodes marks consistent reconstructed nodes; and red nodes are reconstructed with the fallback method. The value following the “P” in the node label shows how much better the chosen solution is in comparison with other possible solution(s). The figure was edited in Adobe Photoshop CS 8.0.

The mapping of the gene boundaries of the ancestral gene order revealed the five plesiomorphy boundaries (A-E) which were represented in the Figs. 5 and 6A. The plesiomorphic block A (*cox1*-*cox2*-*trnK*-*trnD*-*atp8*-*atp6*-*cox3*-*trnG*-*nad3*), B (*trnF*-*nad5*-*trnH*-*nad4*-*nad4L*), D (*rrnL*-*trnV*-*rrnS*) and E (*nad2*-*trnW*) are retained in all taxa of Mygalomorphae and Araneomorphae except family Salticidae (E lost). The block C (*trnP*-*nad6*-*cytb*) is retained in Mygalomorphae and two families of Araneomorphae (Pholcidae and Hypochilidae) (Fig. 6A, Table S5). We have identified 146 derived gene boundaries, including 11 unique boundaries in 16 species. Twenty two derived boundaries were repeated 135 times in 16 species. In spiders, most of the taxa have an identical gene arrangement, though they belong to different families. The gene arrangement of the *P*. *phalangioides* (Pholcidae) is exactly identical to *H*. *thorelli* (Hypochilidae) and *L*. *crotalus* (Theraphosidae) shared 20 boundaries. Further, gene arrangement of other mygalomorphae taxa, *C*. *longitarsis* (Nemesiidae), *O*. *huwena* (Theraphosidae), and *P*. *suthepium* (Dipluridae) are identical and shared 20 boundaries. The gene arrangements of Araneomorhae taxa *O*. *sertatus* (Oxyopidae), *W*. *fidelis* (Lycosidae), *O*. *striatipes* (Thomisidae), *T*. *maxillosa* (Tetragnathidae) *A*. *angulatus*, *N*. *theisi* (Araneidae) are identical and shared 21 boundaries. The derived gene boundaries 1, 7–11, 13, 14, 16, 17 are synapomorphy for Opisthothelae spiders. The gene boundaries 22 and 23 (*rrnS*-*trnI*, *trnI*-*trnQ*) are synapomorphy for the Mygalomorphae + Pholcidae and Hypochilidae clade. Gene boundaries 5, 6 and 12 (*nad6*-*trnI*, *trnI*-*cytb*, *rrnS*-*trnQ*) are synapomorphy for Araneomorphae clade except families Pholcidae and Hypochilidae.

### TreeREx analysis

TreeREx detected 15 nodes, 13 consistent, two inconsistent with four transpositions, seven inversions and one inverse transpositions (Fig. 6B). The transposition of *trnI* at node A14 towards A4 node makes two new gene boundaries, 22 and 23 and become a synapomorphy character for Mygalomorphae and two families of Araneomorphae (Hypochilidae, Pholcidae). The inversion of *trnI* on A3 node towards A2 separated the Mygalomorphae from Pholcidae and Hypochilidae and once more inversion of *trnI* towards *L. crotalus* separated the *L. crotalus* to *O*. *huwena*. Three gene boundaries (5, 6, 12) are synapomorphy for Araneomorphae clade. The inversion of *trnN* on node A10 towards A5 separated the families Dictynidae and Agelenidae from Thomisidae, Lycosidae, Oxyopidae, Selenopidae, and Salticidae. The inverse transposition of *trnN* towards Dictynidae and transposition of (*trnL2*, *trnA*, *trnN*) and inversion of *trnI* towards Agelenidae separated these two families from each other. The inversions of *trnL2* and *trnC* at node A6 towards Selenopidae and transposition of *trnE* and *trnW* towards Salticidae separated these two families from each other.

## Conclusion

The complete mitochondrial genome of endemic giant tarantula *L*. *crotalus* was characterized and compared with other members of the Araneae. The phylogenetic relationships based on mitochondrial genome data are congruent with morphology and the earlier multiple marker gene results. The Araneomorphae and Mygalomorphae are recovered as monophyletic as previously reported. On the other hand, based on morphology, tarantulas (family Theraphosidae) are more inclined to plagiognath condition (chelicerae are intermediate position, neither parallel nor opposing each other). The gene order based analysis of *L*. *crotalus* also indicated the close relationship with Araneomorphae (Hypochilidae and Pholcidae) and other Mygalomorphae species. However, in-depth data on more taxa of Araneae could build up our knowledge towards the rearrangement and evolutionary events.

## Supplementary information

Supplementary Info.
