# Supplementary material for: The Complete Mitochondrial Genome of endemic giant tarantula, Lyrognathus crotalus (Araneae: Theraphosidae) and comparative analysis
Source: Sci Rep. 2020 Jan 9;10:74. doi: 10.1038/s41598-019-57065-8 (PMC6952441; doi:10.1038/s41598-019-57065-8)

**Supplementary Information**

**The Complete Mitochondrial Genome of endemic giant tarantula, *Lyrognathus crotalus* (Araneae: Theraphosidae) and comparative analysis**

Vikas Kumar, Kaomud Tyagi*, Rajasree Chakraborty, Priya Prasad, Shantanu Kundu, Inderjeet Tyagi, Kailash Chandra

Centre for DNA Taxonomy, Molecular Systematics Division, Zoological Survey of India, Kolkata, India

Corresponding author email id: kumud.tyagi5@gmail.com

**Table S1. Details of the Spider mitochondrial genomes accessed from the NCBI GenBank for comparative mitochondrial genomes study.**

| **Sub-order** | **Infra- Order** | **Family** | **Species (Abbreviation)** | **Accession No.** | **Size (bp)** | **GC%** | **PCGs** | **rRNAs** | **tRNAs** | **CR** | **Reference** |
| --- | --- | --- | --- | --- | --- | --- | --- | --- | --- | --- | --- |
| Opisthothelae | Mygalomorphae | Theraphosidae | *Lyrognathous crotalus* (LC) | MN072398 | 13865 | 31.27 | 13 | 2 | 22 | 1 | This study |
|  | Mygalomorphae | Theraphosidae | *Ornithoctonus huwena* (OH) | NC_005925 | 13874 | 30.20 | 13 | 2 | 22 | 1 | [Qiu](https://www.ncbi.nlm.nih.gov/pubmed/?term=Qiu%20Y%5BAuthor%5D&cauthor=true&cauthor_uid=15696368) et al. 2005 |
|  | Mygalomorphae | Dipluridae | *Phyxioschema suthepium* (PS) | JQ407802 | 13931 | 32.60 | 13 | 2 | 22 | 1 | Unpublished GenBank data |
|  | Mygalomorphae | Nemesiidae | *Calisoga longitarsis* (CL) | NC_010780 | 14070 | 36.04 | 13 | 2 | 22 | 1 | Masta & Boore 2008 |
|  | Araneomorphae | Araneidae | *Neoscona theisi* (NT) | NC_026290 | 14156 | 24.84 | 13 | 2 | 22 | 1 | Li el al. 2016 |
|  | Araneomorphae | Araneidae | *Araneus angulatus* (AAN) | NC_032402 | 14205 | 24.86 | 13 | 2 | 22 | 1 | Unpublished GenBank data |
|  | Araneomorphae | Agelenidae | *Agelena silvatica* (AS) | NC_033971 | 14776 | 25.54 | 13 | 2 | 22 | 1 | Zhu & Zhang 2017 |
|  | Araneomorphae | Dictynidae | *Argyroneta aquatica* (AAQ) | NC_026863 | 16000 | 27.79 | 13 | 2 | 22 | 1 | Liu et al. 2014 |
|  | Araneomorphae | Salticidae | *Carrhotus xanthogramma* (CX) | KP402247 | 14563 | 24.95 | 13 | 2 | 22 | 1 | Fang et al. 2016 |
|  | Araneomorphae | Hypochilidae | *Hypochilus thorelli* (HT) | EU523753 | 13999 | 29.66 | 13 | 2 | 22 | 1 | Masta & Boore 2008 |
|  | Araneomorphae | Oxyopidae | *Oxyopes sertatus* (OS) | NC_025224 | 14442 | 24.06 | 13 | 2 | 22 | 1 | Pan et al. 2016 |
|  | Araneomorphae | Thomisidae | *Oxytate striatipes* (OST) | KM507783 | 14407 | 21.79 | 13 | 2 | 22 | 1 | Kim et al. 2016 |
|  | Araneomorphae | Pholcidae | *Pholcus phalangioides* (PP) | JQ407804 | 14459 | 34.14 | 13 | 2 | 22 | 1 | Unpublished GenBank data |
|  | Araneomorphae | Selenopidae | *Selenops bursarius* (SB) | KM114573 | 14272 | 25.60 | 13 | 2 | 22 | 1 | Pan et al. 2016 |
|  | Araneomorphae | Tetragnathidae | *Tetragnatha maxillosa* (TM) | KM504983 | 14414 | 25.50 | 13 | 2 | 22 | 1 | Wang et al. 2016 |
|  | Araneomorphae | Lycosidae | *Wadicosa fidelis* (WF) | KP100666 | 14741 | 23.97 | 13 | 2 | 22 | 1 | Wang et al. 2016 |
| Mesothelae | - | Liphistiidae | *Liphistius erawan* (LE) | JQ407803 | 14197 | 32.28 | 13 | 2 | 22 | 1 | Unpublished GenBank data |

**Table S2. Best model inferred by PartitionFinder for four different dataset used for phylogenetic analysis.**

| Dataset | Best Model | Gene |
| --- | --- | --- |
| PCGs without GBlock (11453 bp) | GTR+I+G | atp6_pos1, cox2_pos1, cytb_pos1, cox3_pos1 |
|  | GTR+I+G | cytb_pos2, cox2_pos2, cox3_pos2, atp6_pos2 |
|  | TRN+I+G | cox1_pos3, cox2_pos3, atp6_pos3, cytb_pos3, cox3_pos3 |
|  | GTR+I+G | atp8_pos2, atp8_pos1, nad3_pos1, nad6_pos1, nad2_pos1 |
|  | TRN+G | nad3_pos3, nad2_pos3, nad6_pos3, atp8_pos3 |
|  | GTR+I+G | cox1_pos1 |
|  | GTR+I+G | cox1_pos2 |
|  | GTR+I+G | nad1_pos1, nad4L_pos1, nad5_pos1, nad4_pos1 |
|  | GTR+I+G | nad1_pos2, nad5_pos2, nad4L_pos2, nad4_pos2 |
|  | TRN+G | nad4L_pos3, nad1_pos3, nad5_pos3, nad4_pos3 |
|  | GTR+I+G | nad3_pos2, nad6_pos2, nad2_pos2 |
| PCGs without GBlock (third codon position excluded) (7640 bp) | GTR+I+G | cox2_pos1, cox3_pos1, cytb_pos1, atp8_pos2, atp6_pos1 |
|  | GTR+I+G | cytb_pos2, cox2_pos2, atp6_pos2, cox3_pos2 |
|  | GTR+I+G | atp8_pos1, nad3_pos1, nad6_pos1, nad2_pos1 |
|  | GTR+I+G | cox1_pos1 |
|  | GTR+I+G | cox1_pos2 |
|  | GTR+I+G | nad1_pos1, nad4Lpos1, nad5_pos1, nad4_pos1 |
|  | GTR+I+G | nad1_pos2, nad5_pos2, nad4L_pos2, nad4_pos2 |
|  | GTR+I+G | nad3_pos2, nad2_pos2, nad6_pos2 |
| PCGs with GBlock (8706 bp) | TVM+I+G | nad3_pos1, nad6_pos1, atp6_pos1, nad2_pos1 |
|  | GTR+I+G | cytb_pos2, cox3_pos2, cox2_pos2, atp8_pos2, nad6_pos2, atp6_pos2, nad3_pos2, nad2_pos2 |
|  | TRN+I+G | atp8_pos1, atp8_pos3, nad6_pos3, atp6_pos3 |
|  | GTR+I+G | cox1_pos1 |
|  | GTR+I+G | cox1_pos2 |
|  | TRN+I+G | cox1_pos3, nad2_pos3, nad3_pos3, cox3_pos3, cytb_pos3, cox2_pos3 |
|  | GTR+I+G | cox2_pos1, cytb_pos1, cox3_pos1 |
|  | GTR+I+G | nad4L_pos1, nad1_pos1, nad5_pos1, nad4_pos1 |
|  | TVM+G | nad1_pos2, nad5_pos2, nad4L_pos2, nad4_pos2 |
|  | TRN+G | nad4L_pos3, nad1_pos3, nad5_pos3, nad4_pos3 |
| PCGs with GBlock (third codon position excluded) (5804 bp) | GTR+I+G | atp8_pos1, nad3_pos1, atp6_pos1, nad2_pos1, nad6_pos1 |
|  | GTR+I+G | cytb_pos2, cox2_pos2, cox3_pos2, atp8_pos2, nad6_pos2, atp6_pos2, nad2_pos2, nad3_pos2 |
|  | GTR+I+G | cox1_pos1 |
|  | GTR+I+G | cox1_pos2 |
|  | GTR+I+G | cox2_pos1, cox3_pos1, cytb_pos1 |
|  | GTR+I+G | nad4L_pos1, nad1_pos1, nad4_pos1, nad5_pos1 |
|  | GTR+G | nad1_pos2, nad5_pos2, nad4_pos2, nad4L_pos2 |

**Table S3. Start and Stop codons of the PCGs of the spider mitochondrial genomes.**

| **Species** |  | ***cox1*** | ***cox2*** | ***cox3*** | ***cytb*** | ***nad1*** | ***nad2*** | ***nad3*** | ***nad4*** | ***nad4L*** | ***nad5*** | ***nad6*** | ***atp6*** | ***atp8*** |
| --- | --- | --- | --- | --- | --- | --- | --- | --- | --- | --- | --- | --- | --- | --- |
| *Lyrognathus crotalus* | Start | ATA | ATG | TTG | ATG | ATA | ATT | ATT | TTG | ATT | ATT | ATT | ATG | ATT |
|  | Stop | TAG | TAG | TAA | TAA | TAA | TAG | TAA | TAA | Not found | TAA | TAA | TAG | TAA |
| *Phyxioschema suthepium* | Start | TTA | TTG | TTG | TTG | ATT | ATT | ATT | ATT | ATC | ATT | TTG | ATG | ATT |
|  | Stop | TAG | TAG | T(AA) | TAA | TAA | TAG | TAG | T(AA) | T(AA) | T(AA) | TA(A) | TAG | TAA |
| *Calisoga longitarsis* | Start | CTA | TTG | TTG | TTG | ATT | TTG | ATT | TTG | ATA | ATT | TTG | ATA | ATA |
|  | Stop | TAG | TAG | T(AA) | T(AA) | TAA | T(AA) | TAG | T(AA) | NOT FOUND | T(AA) | T(AA) | TAG | TAG |
| *Ornithoctonus huwena* | Start | TTA | ATG | TTG | GTG | ATT | ATT | ATA | ATT | ATC | ATC | ATT | ATG | ATG |
|  | Stop | TAG | TAG | T(AA) | TAA | T(AA) | TAA | T(AA) | TAA | NOT FOUND | T(AA) | TAG | TAG | TAA |
| *Neoscona theisi* | Start | CTG | TTG | TTG | ATT | ATT | ATT | ATT | ATA | ATT | ATT | TTG | ATA | ATT |
|  | Stop | TAA | TAG | TAA | TAG | TAG | TAG | TAA | TAA | TAA | T(AA) | TAA | TAA | TAA |
| *Agelena silvatica* | Start | TTA | TTG | TTG | ATT | ATT | ATA | ATT | ATT | ATA | ATA | ATG | ATG | ATA |
|  | Stop | TAA | TAA | TAG | TAA | TAG | TAA | TAA | T(AA) | NOT FOUND | T(AA) | TAA | TAA | TAG |
| *Argyroneta aquatica* | Start | TTG | TTG | TTG | ATT | ATT | ATT | ATT | TTG | ATT | ATT | TTG | ATG | ATC |
|  | Stop | TAA | TAG | TAG | TAA | TAG | T(AA) | T(AA) | T(AA) | NOT FOUND | T(AA) | TAG | TAA | TAA |
| *Hypochilus thorelli* | Start | ATA | TTG | ATT | TTG | ATA | ATA | ATA | ATT | ATT | ATT | ATT | ATG | ATT |
|  | Stop | T(AA) | TAG | TAA | TAA | TAG | TAG | TAA | T(AA) | NOT FOUND | T(AA) | T(AA) | TAA | TAA |
| *Wadicosa fidelis* | Start | ATA | TTG | TTG | ATT | ATA | ATT | ATA | TTG | ATT | ATT | TTG | ATA | ATA |
|  | Stop | T(AA) | TAA | TAA | TAA | T(AA) | TAG | TAA | TA(A) | TAA | T(AA) | TAA | TAA | TAA |
| *Oxyopes sertatus* | Start | ATA | TTG | TTG | ATT | ATA | ATT | ATT | TTG | ATG | ATA | GTG | ATA | ATT |
|  | Stop | T(AA) | TAA | TAA | TA(A) | T(AA) | TAG | TAA | TAA | NOT FOUND | TA(A) | TAA | TAA | TAA |
| *Pholcus phalangioides* | Start | ATA | GTG | ATG | ATG | ATT | GTG | ATT | ATG | ATA | ATA | ATG | GTG | ATT |
|  | Stop | TAG | T(AA) | TAA | TAG | TAG | TAA | TAG | TAA | TAA | T(AA) | TAA | TAA | TAA |
| *Carrhotus xanthogramma* | Start | TTA | TTG | ATG | ATT | ATT | ATA | ATA | ATG | ATA | ATT | ATA | ATA | ATT |
|  | Stop | TAA | T(AA) | T(AA) | TAA | TAG | TAA | TAA | T(AA) | TAA | TAA | TAA | TAA | TAA |
| *Selenops bursarius* | Start | TTA | GTG | TTG | ATT | ATC | ATT | ATT | TTG | ATT | TTG | ATT | ATA | ATA |
|  | Stop | T(AA) | TAA | TAA | T(AA) | TAG | TAA | TAA | TAA | NOT FOUND | TAA | T(AA) | TAA | TAA |
| *Oxytate striatipes* | Start | TTA | TTG | TTG | ATT | ATT | ATC | ATT | TTG | ATT | AAT | ATT | ATA | ATT |
|  | Stop | T(AA) | TAG | TAA | T(AA) | TAG | TAA | TAA | T(AA) | NOT FOUND | T(AA) | TAA | TAA | TAA |
| *Tetragnatha maxillosa* | Start | TTT | TTG | TTG | ATT | ATA | ATT | ATA | ATA | ATT | ATA | ATA | ATA | ATT |
|  | Stop | TAA | TAG | TAA | T(AA) | TAA | T(AA) | TAA | T(AA) | NOT FOUND | TAA | TAA | TAA | TAA |
| *Liphistius erawan* | Start | CTG | ATG | TTG | ATG | ATA | ATG | ATT | ATG | ATG | ATT | ATA | ATG | ATC |
|  | Stop | TAA | T(AA) | T(AA) | TAA | TAG | T(AA) | TAG | TAA | TAA | TAA | TAA | TAA | TAA |
| *Limulus polyphemus* | Start | TTA | ATG | ATG | ATG | TTG | ATC | ATT | ATG | ATG | TTG | ATC | ATG | ATT |
|  | Stop | TAA | T(AA) | T(AA) | TAA | TAA | T(AA) | TAA | TAG | TAG | T(AA) | TAA | TAA | TAA |
| *Araneus angulatus* | Start | TTA | TTG | TTG | ATT | ATA | ATT | ATA | ATA | ATA | AAT | TTG | ATA | ATT |
|  | Stop | TAA | TAA | TAG | TAA | TAA | TAG | TAA | T(AA) | T(AA) | TAA | T(AA) | TAA | TAA |

**Table S4. RSCU analysis of the PCGs of *L. crotalus* mitochondrial genome.**

| **Amino acid** | **Codon** | **Number** | **Frequency (%)** | **RSCU** | **Amino acid** | **Codon** | **Number** | **Frequency (%)** | **RSCU** |
| --- | --- | --- | --- | --- | --- | --- | --- | --- | --- |
| Phenylalanine | UUU | 229 | 6.39 | 1.78 | Tyrosine | UAU | 144 | 4.02 | 1.73 |
|  | UUC | 29 | 0.81 | 0.22 |  | UAC | 22 | 0.61 | 0.27 |
| Leucine 2 | UUA | 160 | 4.47 | 3.23 | Stop codon | UAA(*) | 93 | 2.60 | 1.18 |
|  | UUG | 94 | 2.62 | 1.9 |  | UAG(*) | 65 | 1.81 | 0.82 |
| Leucine 1 | CUU | 20 | 0.56 | 0.4 | Histidine | CAU | 45 | 1.26 | 1.76 |
|  | CUC | 5 | 0.14 | 0.1 |  | CAC | 6 | 0.17 | 0.24 |
|  | CUA | 11 | 0.31 | 0.22 | Glutamine | CAA | 27 | 0.75 | 1.35 |
|  | CUG | 7 | 0.20 | 0.14 |  | CAG | 13 | 0.36 | 0.65 |
| Isoleucine | AUU | 191 | 5.33 | 1.8 | Asparagine | AAU | 178 | 4.97 | 1.77 |
|  | AUC | 21 | 0.59 | 0.2 |  | AAC | 23 | 0.64 | 0.23 |
| Methionine | AUA | 149 | 4.16 | 1.32 | Lysine | AAA | 124 | 3.46 | 1.19 |
|  | AUG | 77 | 2.15 | 0.68 |  | AAG | 85 | 2.37 | 0.81 |
| Valine | GUU | 133 | 3.71 | 1.69 | Aspartic acid | GAU | 97 | 2.71 | 1.8 |
|  | GUC | 9 | 0.25 | 0.11 |  | GAC | 11 | 0.31 | 0.2 |
|  | GUA | 93 | 2.60 | 1.18 | Glutamic acid | GAA | 88 | 2.46 | 1.17 |
|  | GUG | 80 | 2.23 | 1.02 |  | GAG | 62 | 1.73 | 0.83 |
| Serine1 | UCU | 103 | 2.88 | 2.01 | Cysteine | UGU | 32 | 0.89 | 0.98 |
|  | UCC | 27 | 0.75 | 0.53 |  | UGC | 33 | 0.92 | 1.02 |
|  | UCA | 41 | 1.14 | 0.8 | Tryptophan | UGA | 105 | 2.93 | 1.34 |
|  | UCG | 18 | 0.50 | 0.35 |  | UGG | 52 | 1.45 | 0.66 |
| Proline | CCU | 54 | 1.51 | 2.16 | Arginine | CGU | 11 | 0.31 | 1.16 |
|  | CCC | 22 | 0.61 | 0.88 |  | CGC | 2 | 0.06 | 0.21 |
|  | CCA | 19 | 0.53 | 0.76 |  | CGA | 14 | 0.39 | 1.47 |
|  | CCG | 5 | 0.14 | 0.2 |  | CGG | 11 | 0.31 | 1.16 |
| Threonine | ACU | 45 | 1.26 | 2 | Serine2 | AGU | 28 | 0.78 | 0.55 |
|  | ACC | 9 | 0.25 | 0.4 |  | AGC | 24 | 0.67 | 0.47 |
|  | ACA | 29 | 0.81 | 1.29 |  | AGA | 128 | 3.57 | 2.5 |
|  | ACG | 7 | 0.20 | 0.31 |  | AGG | 40 | 1.12 | 0.78 |
| Alanine | GCU | 52 | 1.45 | 2.39 | Glycine | GGU | 57 | 1.59 | 0.93 |
|  | GCC | 7 | 0.20 | 0.32 |  | GGC | 19 | 0.53 | 0.31 |
|  | GCA | 11 | 0.31 | 0.51 |  | GGA | 91 | 2.54 | 1.49 |
|  | GCG | 17 | 0.47 | 0.78 |  | GGG | 78 | 2.18 | 1.27 |

**Table S5**. Nucleotide composition and skewness in different mitochondrial locus of Spider mitochondrial genomes.

| **Species** | **Size(bp)** | **A%** | **G%** | **T%** | **C%** | **AT%** | **GC%** | **At skew** | **Gc skew** |
| --- | --- | --- | --- | --- | --- | --- | --- | --- | --- |
| **Whole mtgenome** | | | | | | | | | |
| *Lyrognathus crotalus* | 13866.0 | 31.96 | 21.21 | 36.77 | 10.05 | 68.74 | 31.26 | -0.01 | 0.15 |
| *Phyxioschema suthepium* | 13931.0 | 32.35 | 23.99 | 35.06 | 8.61 | 67.40 | 32.60 | -0.04 | 0.47 |
| *Calisoga longitarsis* | 14068.0 | 27.30 | 24.59 | 36.66 | 11.44 | 63.96 | 36.04 | -0.15 | 0.36 |
| *Ornithoctonus huwena* | 13874.0 | 31.99 | 20.29 | 37.81 | 9.91 | 69.80 | 30.20 | -0.08 | 0.34 |
| *Neoscona theisi* | 14156.0 | 35.37 | 15.56 | 39.79 | 9.29 | 75.16 | 24.84 | -0.06 | 0.25 |
| *Argyroneta aquatica* | 15932.0 | 31.28 | 19.01 | 40.93 | 8.79 | 72.21 | 27.79 | -0.13 | 0.37 |
| *Wadicosa fidelis* | 14741.0 | 33.06 | 15.72 | 42.96 | 8.25 | 76.03 | 23.97 | -0.13 | 0.31 |
| *Oxyopes sertatus* | 14442.0 | 33.05 | 15.90 | 42.89 | 8.16 | 75.94 | 24.06 | -0.13 | 0.32 |
| *Selenops bursarius* | 14272.0 | 32.62 | 16.91 | 41.77 | 8.70 | 74.40 | 25.60 | -0.12 | 0.32 |
| *Oxytate striatipes* | 14407.0 | 35.81 | 13.20 | 42.40 | 8.59 | 78.21 | 21.79 | -0.08 | 0.21 |
| *Tetragnatha maxillosa* | 14414.0 | 34.07 | 15.92 | 40.43 | 9.58 | 74.50 | 25.50 | -0.09 | 0.25 |
| *Hypochilus thorelli* | 13990.0 | 30.23 | 18.77 | 40.11 | 10.89 | 70.34 | 29.66 | -0.14 | 0.27 |
| *Pholcus phalangioides* | 14459.0 | 26.65 | 23.40 | 39.21 | 10.74 | 65.86 | 34.14 | -0.19 | 0.37 |
| *Carrhotus xanthogramma* | 14563.0 | 34.18 | 15.72 | 40.88 | 9.23 | 75.05 | 24.95 | -0.09 | 0.26 |
| *Agelena silvatica* | 14776.0 | 31.17 | 16.63 | 43.29 | 8.91 | 74.46 | 25.54 | -0.16 | 0.30 |
| *Araneus angulatus* | 14205.0 | 35.34 | 15.49 | 39.80 | 9.37 | 75.14 | 24.86 | -0.06 | 0.25 |
| *Liphtius erawan* | 14197.0 | 34.68 | 10.32 | 33.04 | 21.96 | 67.72 | 32.28 | 0.02 | -0.36 |
| *Limulus polyphemus* | 14985.0 | 37.52 | 9.74 | 30.05 | 22.69 | 67.57 | 32.43 | 0.11 | -0.40 |
| **Protein Coding Genes** | | | | | | | | | |
| *Lyrognathus crotalus* | 10587 | 31.67 | 21.85 | 37.05 | 9.44 | 68.72 | 31.28 | -0.08 | 0.40 |
| *Phyxioschema suthepium* | 10730 | 30.96 | 25.46 | 35.63 | 7.95 | 66.59 | 33.41 | -0.07 | 0.52 |
| *Calisoga longitarsis* | 10736 | 25.88 | 25.93 | 37.25 | 10.94 | 63.13 | 36.87 | -0.18 | 0.41 |
| *Ornithoctonus huwena* | 10724 | 31.13 | 21.01 | 38.64 | 9.22 | 69.77 | 30.23 | -0.11 | 0.39 |
| *Neoscona theisi* | 10807 | 34.23 | 16.67 | 40.11 | 8.99 | 74.34 | 25.66 | -0.08 | 0.30 |
| *Argyroneta aquatica* | 10787 | 29.08 | 21.45 | 40.73 | 8.73 | 69.82 | 30.18 | -0.17 | 0.42 |
| *Wadicosa fidelis* | 10850 | 32.23 | 16.20 | 43.51 | 8.06 | 75.74 | 24.26 | -0.15 | 0.34 |
| *Oxyopes sertatus* | 10777 | 32.28 | 16.99 | 42.71 | 8.02 | 74.99 | 25.01 | -0.14 | 0.36 |
| *Selenops bursarius* | 10756 | 31.80 | 17.94 | 42.02 | 8.24 | 73.82 | 26.18 | -0.14 | 0.37 |
| *Oxytate striatipes* | 10784 | 35.30 | 13.86 | 42.40 | 8.44 | 77.70 | 22.30 | -0.09 | 0.24 |
| *Tetragnatha maxillosa* | 10714 | 32.87 | 17.15 | 40.58 | 9.40 | 73.46 | 26.54 | -0.10 | 0.29 |
| *Hypochilus thorelli* | 10752 | 28.96 | 20.29 | 40.05 | 10.70 | 69.01 | 30.99 | -0.16 | 0.31 |
| *Pholcus phalangioides* | 10631 | 25.37 | 24.61 | 40.00 | 10.03 | 65.37 | 34.63 | -0.22 | 0.42 |
| *Carrhotus xanthogramma* | 10809 | 32.89 | 17.01 | 41.19 | 8.91 | 74.08 | 25.92 | -0.11 | 0.31 |
| *Agelena silvatica* | 10642 | 29.67 | 18.01 | 43.85 | 8.46 | 73.53 | 26.47 | -0.19 | 0.36 |
| *Araneus angulatus* | 10747 | 34.17 | 16.55 | 40.24 | 9.04 | 74.41 | 25.59 | -0.08 | 0.29 |
| *Liphtius erawan* | 10794 | 34.52 | 10.29 | 32.22 | 22.97 | 66.74 | 33.26 | 0.03 | -0.38 |
| *Limulus polyphemus* | 11077 | 37.00 | 9.64 | 29.42 | 23.93 | 66.43 | 33.57 | 0.11 | -0.43 |
| **tRNAs** | | | | | | | | | |
| *Lyrognathus crotalus* | 1459 | 34.41 | 19.12 | 34.75 | 11.72 | 69.16 | 30.84 | -0.005 | 0.24 |
| *Phyxioschema suthepium* | 1188 | 34.93 | 21.89 | 33.67 | 9.51 | 68.60 | 31.40 | 0.02 | 0.39 |
| *Calisoga longitarsis* | 1160 | 31.98 | 20.78 | 35.09 | 12.16 | 67.07 | 32.93 | -0.05 | 0.26 |
| *Ornithoctonus huwena* | 1240 | 36.29 | 19.76 | 33.87 | 10.08 | 70.16 | 29.84 | 0.03 | 0.32 |
| *Neoscona theisi* | 1323 | 38.40 | 13.08 | 38.40 | 10.13 | 76.80 | 23.20 | 0.00 | 0.13 |
| *Argyroneta aquatica* | 1325 | 36.98 | 13.89 | 39.85 | 9.28 | 76.83 | 23.17 | -0.04 | 0.20 |
| *Wadicosa fidelis* | 1208 | 37.50 | 15.31 | 38.25 | 8.94 | 75.75 | 24.25 | -0.01 | 0.26 |
| **Species** | **Size(bp)** | **A%** | **G%** | **T%** | **C%** | **AT%** | **GC%** | **At skew** | **Gc skew** |
| *Oxyopes sertatus* | 1149 | 37.25 | 15.93 | 37.16 | 9.66 | 74.41 | 25.59 | 0.00 | 0.24 |
| *Selenops bursarius* | 1267 | 36.86 | 13.81 | 41.52 | 7.81 | 78.37 | 21.63 | -0.06 | 0.28 |
| *Oxytate striatipes* | 1146 | 36.91 | 14.92 | 39.53 | 8.64 | 76.44 | 23.56 | -0.03 | 0.27 |
| *Tetragnatha maxillosa* | 1214 | 31.14 | 22.24 | 36.00 | 10.63 | 67.13 | 32.87 | -0.07 | 0.35 |
| *Hypochilus thorelli* | 1324 | 36.86 | 15.03 | 37.54 | 10.57 | 74.40 | 25.60 | -0.01 | 0.17 |
| *Pholcus phalangioides* | 1315 | 36.50 | 15.82 | 38.33 | 9.35 | 74.83 | 25.17 | -0.02 | 0.26 |
| *Carrhotus xanthogramma* | 1146 | 39.79 | 12.74 | 39.70 | 7.77 | 79.49 | 20.51 | 0.00 | 0.24 |
| *Agelena silvatica* | 1182 | 38.07 | 11.34 | 40.69 | 9.90 | 78.76 | 21.24 | -0.03 | 0.07 |
| *Araneus angulatus* | 1192 | 38.26 | 12.75 | 39.09 | 9.90 | 77.35 | 22.7 | - .01 | .13 |
| *Liphtius erawan* | 1266 | 35.07 | 12.88 | 34.12 | 17.93 | 69.19 | 30.81 | 0.01 | -0.16 |
| *Limulus polyphemus* | 1468 | 37.26 | 12.13 | 31.40 | 19.21 | 68.66 | 31.34 | 0.09 | -0.23 |
| **rRNA** | | | | | | | | | |
| *Lyrognathus crotalus* | 1756 | 30.92 | 19.08 | 37.76 | 12.24 | 68.68 | 31.32 | -0.10 | 0.22 |
| *Phyxioschema suthepium* | 1624 | 37.99 | 17.18 | 33.25 | 11.58 | 71.24 | 28.76 | 0.07 | 0.19 |
| *Calisoga longitarsis* | 1753 | 32.12 | 19.28 | 34.68 | 13.92 | 66.80 | 33.20 | -0.04 | 0.16 |
| *Ornithoctonus huwena* | 1714 | 34.36 | 16.57 | 35.94 | 13.13 | 70.30 | 29.70 | -0.02 | 0.12 |
| *Neoscona theisi* | 1723 | 38.89 | 11.09 | 38.94 | 11.09 | 77.83 | 22.17 | 0.00 | 0.00 |
| *Argyroneta aquatica* | 1725 | 33.74 | 11.19 | 45.91 | 9.16 | 79.65 | 20.35 | -0.15 | 0.10 |
| *Wadicosa fidelis* | 1746 | 35.45 | 12.43 | 42.10 | 10.02 | 77.55 | 22.45 | -0.09 | 0.11 |
| *Oxyopes sertatus* | 1729 | 33.14 | 12.72 | 42.16 | 11.97 | 75.30 | 24.70 | -0.12 | 0.03 |
| *Selenops bursarius* | 1716 | 37.00 | 9.32 | 44.76 | 8.92 | 81.76 | 18.24 | -0.09 | 0.02 |
| *Oxytate striatipes* | 1718 | 36.38 | 10.13 | 44.41 | 9.08 | 80.79 | 19.21 | -0.10 | 0.05 |
| *Tetragnatha maxillosa* | 1695 | 29.03 | 17.82 | 40.53 | 12.63 | 69.56 | 30.44 | -0.17 | 0.17 |
| *Hypochilus thorelli* | 1692 | 38.42 | 10.11 | 41.73 | 9.75 | 80.14 | 19.86 | -0.04 | 0.02 |
| *Pholcus phalangioides* | 1706 | 35.64 | 12.72 | 42.20 | 9.44 | 77.84 | 22.16 | -0.08 | 0.15 |
| *Carrhotus xanthogramma* | 1888 | 37.45 | 9.32 | 44.23 | 9.00 | 81.67 | 18.33 | -0.08 | 0.02 |
| *Agelena silvatica* | 1700 | 38.59 | 10.65 | 41.41 | 9.35 | 80.00 | 20.00 | -0.04 | 0.06 |
| *Araneus angulatus* | 1719 | 39.1 | 10.4 | 40.5 | 9.9 | 79.64 | 20.4 | - .02 | .02 |
| *Liphtius erawan* | 1832 | 35.53 | 9.50 | 35.15 | 19.81 | 70.69 | 29.31 | 0.01 | -0.35 |
| *Limulus polyphemus* | 2095 | 40.43 | 8.83 | 30.07 | 20.67 | 70.50 | 29.50 | 0.15 | -0.40 |
| **Control Region** | | | | | | | | | |
| *Lyrognathus crotalus* | 365 | 35.07 | 18.63 | 32.60 | 13.70 | 67.67 | 32.33 | 0.04 | 0.15 |
| *Phyxioschema suthepium* | 387 | 39.28 | 18.86 | 32.04 | 9.82 | 71.32 | 28.68 | 0.10 | 0.32 |
| *Calisoga longitarsis* | 478 | 33.05 | 21.97 | 33.26 | 11.72 | 66.32 | 33.68 | 0.00 | 0.30 |
| *Ornithoctonus huwena* | 396 | 38.38 | 20.20 | 29.80 | 11.62 | 68.18 | 31.82 | 0.13 | 0.27 |
| *Neoscona theisi* | 559 | 41.68 | 12.34 | 37.92 | 8.05 | 79.61 | 20.39 | 0.05 | 0.21 |
| *Argyroneta aquatica* | 1983 | 37.57 | 13.46 | 40.65 | 8.32 | 78.21 | 21.79 | -0.04 | 0.24 |
| *Wadicosa fidelis* | 1071 | 32.40 | 23.34 | 35.48 | 8.78 | 67.88 | 32.12 | -0.05 | 0.45 |
| *Oxyopes sertatus* | 839 | 31.35 | 14.90 | 46.36 | 7.39 | 77.71 | 22.29 | -0.19 | 0.34 |
| *Selenops bursarius* | 630 | 35.08 | 9.68 | 44.13 | 11.11 | 79.21 | 20.79 | -0.11 | -0.07 |
| *Oxytate striatipes* | 489 | 31.49 | 16.97 | 39.06 | 12.47 | 70.55 | 29.45 | -0.11 | 0.15 |
| *Tetragnatha maxillosa* | 829 | 35.83 | 15.56 | 37.39 | 11.22 | 73.22 | 26.78 | -0.02 | 0.16 |
| *Hypochilus thorelli* | 503 | 39.76 | 12.92 | 37.57 | 9.74 | 77.34 | 22.66 | 0.03 | 0.14 |
| *Pholcus phalangioides* | 1076 | 32.25 | 21.00 | 32.16 | 14.59 | 64.41 | 35.59 | 0.00 | 0.18 |
| *Carrhotus xanthogramma* | 900 | 39.89 | 10.33 | 40.11 | 9.67 | 80.00 | 20.00 | 0.00 | 0.03 |
| *Agelena silvatica* | 599 | 36.73 | 10.85 | 44.24 | 8.18 | 80.97 | 19.03 | -0.09 | 0.14 |
| *Araneus angulatus* | 649 | 39.45 | 15.72 | 31.90 | 12.94 | 71.34 | 28.66 | .11 | .10 |
| *Liphtius erawan* | 338 | 35.21 | 9.17 | 42.90 | 12.72 | 78.11 | 21.89 | -0.10 | -0.16 |
| *Limulus polyphemus* | 348 | 38.79 | 8.62 | 42.53 | 10.06 | 81.32 | 18.68 | -0.05 | -0.08 |

**Table S6. Input file for CREx and TreeREx analysis using gene order of spiders for phylogenetic relationships.**

> *Limulus polyphemus*

cox1 cox2 K D atp8 atp6 cox3 G nad3 A R N S1 E -F -nad5 -H -nad4 -nad4l T -P nad6 cytb S2 -nad1 -L2 -L1 -rrnL -V -rrnS CR I -Q M nad2 W -C -Y

> *Phyxioschema suthepium*

cox1 cox2 K D atp8 atp6 cox3 G nad3 -L2 N A S1 R E -F -nad5 -H -nad4 -nad4l -P nad6 cytb S2 T -nad1 -L1 -rrnL -V -rrnS -I -Q CR M nad2 W -Y -C

>*Lyrognathus crotalus*

cox1 cox2 K D atp8 atp6 cox3 G nad3 -L2 N A S1 R E -F -nad5 -H -nad4 -nad4l -P nad6 cytb S2 T -nad1 -L1 -rrnL -V -rrnS I -Q CR M nad2 W -Y -C

>*Calisoga longitarsis*

cox1 cox2 K D atp8 atp6 cox3 G nad3 -L2 N A S1 R E -F -nad5 -H -nad4 -nad4l -P nad6 cytb S2 T -nad1 -L1 -rrnL -V -rrnS -I -Q CR M nad2 W -Y -C

> *Ornithoctonus huwena*

cox1 cox2 K D atp8 atp6 cox3 G nad3 -L2 N A S1 R E -F -nad5 -H -nad4 -nad4l -P nad6 cytb S2 T -nad1 -L1 -rrnL -V -rrnS -I -Q CR M nad2 W -Y -C

> *Hypochilus thorelli*

cox1 cox2 K D atp8 atp6 cox3 G nad3 -L2 N A S1 R E -F -nad5 -H -nad4 -nad4l -P nad6 cytb S2 T -nad1 -L1 -rrnL -V -rrnS I -Q CR M nad2 W -Y -C

> *Pholcus phalangioides*

cox1 cox2 K D atp8 atp6 cox3 G nad3 -L2 N A S1 R E -F -nad5 -H -nad4 -nad4l -P nad6 cytb S2 T -nad1 -L1 -rrnL -V -rrnS I -Q CR M nad2 W -Y -C

> *Neoscona theisi*

cox1 cox2 K D atp8 atp6 cox3 G nad3 -L2 N A S1 R E -F -nad5 -H -nad4 -nad4l -P nad6 I cytb S2 T -nad1 -L1 -rrnL -V -rrnS -Q CR M nad2 W -Y -C

>*Oxytate striatipes*

cox1 cox2 K D atp8 atp6 cox3 G nad3 -L2 N A S1 R E -F -nad5 -H -nad4 -nad4l -P nad6 I cytb S2 T -nad1 -L1 -rrnL -V -rrnS -Q CR M nad2 W -Y -C

> *Araneus angulatus*

cox1 cox2 K D atp8 atp6 cox3 G nad3 -L2 N A S1 R E -F -nad5 -H -nad4 -nad4l -P nad6 I cytb S2 T -nad1 -L1 -rrnL -V -rrnS -Q CR M nad2 W -Y -C

> *Selenops bursarius*

cox1 cox2 K D atp8 atp6 cox3 G nad3 L2 N A S1 R E -F -nad5 -H -nad4 -nad4l -P nad6 I cytb S2 T -nad1 -L1 -rrnL -V -rrnS -Q CR M nad2 W -Y -C

>*Oxyopes sertatus*

cox1 cox2 K D atp8 atp6 cox3 G nad3 -L2 N A S1 R E -F -nad5 -H -nad4 -nad4l -P nad6 I cytb S2 T -nad1 -L1 -rrnL -V -rrnS -Q CR M nad2 W -Y -C

> *Wadicosa fidelis*

cox1 cox2 K D atp8 atp6 cox3 G nad3 -L2 N A S1 R E -F -nad5 -H -nad4 -nad4l -P nad6 I cytb S2 T -nad1 -L1 -rrnL -V -rrnS -Q CR M nad2 W -Y -C

> *Argyroneta aquatica*

cox1 cox2 K D atp8 atp6 cox3 G nad3 -L2 -N A S1 R E -F -nad5 -H -nad4 -nad4l -P nad6 I cytb S2 T -nad1 -L1 -rrnL -V -rrnS -Q CR M nad2 W -Y -C

> *Agelena silvatica*

cox1 cox2 K D atp8 atp6 cox3 G nad3 S1 R E -L2 A N -F -nad5 -H -nad4 -nad4l -P nad6 -I cytb S2 T -nad1 -L1 -rrnL -V -rrnS -Q CR M nad2 W -Y -C

> *Carrhotus xanthogramma*

cox1 cox2 K D atp8 atp6 cox3 G nad3 -L2 N E A S1 R -F -nad5 -H -nad4 -nad4l W -P nad6 I cytb S2 T -nad1 -L1 -rrnL -V -rrnS -Q CR M nad2 -Y -C

> *Tetragnatha maxillosa*

cox1 cox2 K D atp8 atp6 cox3 G nad3 -L2 N A S1 R E -F -nad5 -H -nad4 -nad4l -P nad6 I cytb S2 T -nad1 -L1 -rrnL -V -rrnS -Q CR M nad2 W -Y -C

> *Liphistius erawan*

cox1 cox2 K D atp8 atp6 cox3 G nad3 A R N S1 E -F -nad5 -H -nad4 -nad4l T -P nad6 cytb S2 -nad1 -L2 -L1 -rrnL -V -rrnS CR I -Q M nad2 W -C –Y

**Table S7. Derived Gene Boundaries (DGB) identified in 17 spider species using gene order. Species names were abbreviated as per Table S1.**

| **DGB** | **AS** | **DGB** | **PS/OH/CL** | **DGB** | **PP/HT/LC** | **DGB** | **OST/OS/NT/**  **TM/WF/AAN** | **DGB** | **AAQ** | **DGB** | **SB** | **DGB** | **CX** |
| --- | --- | --- | --- | --- | --- | --- | --- | --- | --- | --- | --- | --- | --- |
| *cox1*-*cox2* |  | *cox1*-*cox2* |  | *cox1*-*cox2* |  | *cox1*-*cox2* |  | *cox1*-*cox2* |  | *cox1*-*cox2* |  | *cox1*-*cox2* |  |
| *cox2*-*K* |  | *cox2*-*K* |  | *cox2*-*K* |  | *cox2*-*K* |  | *cox2*-*K* |  | *cox2*-*K* |  | *cox2*-*K* |  |
| *K*-*D* |  | *K*-*D* |  | *K*-*D* |  | *K*-*D* |  | *K*-*D* |  | *K*-*D* |  | *K*-*D* |  |
| *D*-*atp8* |  | *D*-*atp8* |  | *D*-*atp8* |  | *D*-*atp8* |  | *D*-*atp8* |  | *D*-*atp8* |  | *D*-*atp8* |  |
| *atp8*-*atp6* |  | *atp8*-*atp6* |  | *atp8*-*atp6* |  | *atp8*-*atp6* |  | *atp8*-*atp6* |  | *atp8*-*atp6* |  | *atp8*-*atp6* |  |
| *atp6*-*cox3* |  | *atp6*-*cox3* |  | *atp6*-*cox3* |  | *atp6*-*cox3* |  | *atp6*-*cox3* |  | *atp6*-*cox3* |  | *atp6*-*cox3* |  |
| *cox3*-*G* |  | *cox3*-*G* |  | *cox3*-*G* |  | *cox3*-*G* |  | *cox3*-*G* |  | *cox3*-*G* |  | *cox3*-*G* |  |
| *G*-*nad3* |  | *G*-*nad3* |  | *G*-*nad3* |  | *G*-*nad3* |  | *G*-*nad3* |  | *G*-*nad3* |  | *G*-*nad3* |  |
| *nad3*-*S1* |  | *nad3*-*L2* | 18 | *nad3*- *L2* | 18 | *nad3*- *L2* | 18 | *nad3*- *L2* | 18 | *nad3*- *L2* | 18 | *nad3*- *L2* | 18 |
| *S1*-*R* | 1 | *L2*-*N* | 19 | *L2*-*N* | 19 | *L2*-*N* | 19 | *L2*-*N* | 19 | *L2*-*N* | 19 | *L2*-*N* | 19 |
| *R*-*E* | 2 | *N*-*A* | 3 | *N*-*A* | 3 | *N*-*A* | 3 | *N*-*A* | 3 | *N*-*A* | 3 | *N*-*E* |  |
| *E*-*L2* |  | *A*-*S1* | 20 | *A*-*S1* | 20 | *A*-*S1* | 20 | *A*-*S1* | 20 | *A*-*S1* | 20 | *E*-*A* |  |
| *L2*-*A* |  | *S1*-*R* | 1 | *S1*-*R* | 1 | *S1*-*R* | 1 | *S1*-*R* | 1 | *S1*-*R* | 1 | *A*-*S1* | 20 |
| *A*-*N* | 3 | *R*-*E* | 2 | *R*-*E* | 2 | *R*-*E* | 2 | *R*-*E* | 2 | *R*-*E* | 2 | *S1*-*R* | 1 |
| *N*-*F* |  | *E*-*F* | 21 | *E*-*F* | 21 | *E*-*F* | 21 | *E*-*F* | 21 | *E*-*F* | 21 | *R*-*F* |  |
| *F*-*nad5* |  | *F*- *nad5* |  | *F*- *nad5* |  | *F*- *nad5* |  | *F*- *nad5* |  | *F*- *nad5* |  | *F*- *nad5* |  |
| *nad5*-*H* |  | *nad5*-*H* |  | *nad5*-*H* |  | *nad5*-*H* |  | *nad5*-*H* |  | *nad5*-*H* |  | *nad5*-*H* |  |
| *H*- *nad4* |  | *H*- *nad4* |  | *H*-*nad4* |  | *H*-*nad4* |  | *H*- *nad4* |  | *H*- *nad4* |  | *H*- *nad4* |  |
| *nad4*- *nad4L* |  | *nad4*-*nad4L* |  | *nad4*-*nad4L* |  | *nad4*-*nad4L* |  | *nad4*-*nad4L* |  | *nad4*-*nad4L* |  | *nad4*-*nad4L* |  |
| *nad4L*-*P* | 4 | *nad4L*-*P* | 4 | *nad4L* -*P* | 4 | *nad4L* -*P* | 4 | *nad4L* -*P* | 4 | *nad4L* -*P* | 4 | *nad4L* -*W* |  |
| *P*-*nad6* |  | *P*-*nad6* |  | *P*- *nad6* |  | *P*- *nad6* |  | *P*- *nad6* |  | *P*- *nad6* |  | *W*-*P* |  |
| *nad6*-*I* | 5 | *nad6*- *cytb* |  | *nad6*- *cytb* |  | *nad6*-*I* | 5 | *nad6*-*I* | 5 | *nad6*-*I* | 5 | *P*- *nad6* |  |
| *I*-*cytb* | 6 | *cytb* -*S2* | 7 | *cytb* -*S2* | 7 | *I*- *cytb* | 6 | *I*- *cytb* | 6 | *I*- *cytb* | 6 | *nad6*-*I* | 5 |
| *cytb*-*S2* | 7 | *S2*-*T* | 8 | *S2*-*T* | 8 | *cytb* -*S2* | 7 | *cytb*-*S2* | 7 | *cytb*-*S2* | 7 | *I*-*cytb* | 6 |
| *S2*-*T* | 8 | *T*- *nad1* | 9 | *T*- *nad1* | 9 | *S2*-*T* | 8 | *S2*-*T* | 8 | *S2*-*T* | 8 | *cytb*-*S2* | 7 |
| *T*-*nad1* | 9 | *nad1*-*L1* | 10 | *nad1*-*L1* | 10 | *T*- *nad1* | 9 | *T*- *nad1* | 9 | *T*- *nad1* | 9 | *S2*-*T* | 8 |
| *nad1*-*L1* | 10 | *L1*- *rrnL* | 11 | *L1*- *rrnL* | 11 | *nad1*-*L1* | 10 | *nad1*-*L1* | 10 | *nad1*-*L1* | 10 | *T*- *nad1* | 9 |
| *L1*-*rrnL* | 11 | *rrnL* -*V* |  | *rrnL* -*V* |  | *L1*- *rrnL* | 11 | *L1*- *rrnL* | 11 | *L1*- *rrnL* | 11 | *nad1*-*L1* | 10 |
| *rrnL*-*V* |  | *V*- *rrnS* |  | *V*- *rrnS* |  | *rrnL* -*V* |  | *rrnL* -*V* |  | *rrnL* -*V* |  | *L1*- *rrnL* | 11 |
| *V*- *rrnS* |  | *rrnS* -*I* | 22 | *rrnS* -*I* | 22 | *V*- *rrnS* |  | *V*- *rrnS* |  | *V*- *rrnS* |  | *rrnL* -*V* |  |
| *rrnS* -*Q* | 12 | *I*-*Q* | 23 | *I*-*Q* | 23 | *rrnS* -*Q* | 12 | *rrnS* -*Q* | 12 | *rrnS* -*Q* | 12 | *V*- *rrnS* |  |
| *Q*-*M* | 13 | *Q*-*M* | 13 | *Q*-*M* | 13 | *Q*-*M* | 13 | *Q*-*M* | 13 | *Q*-*M* | 13 | *rrnS*-*Q* | 12 |
| *M* -*nad2* | 14 | *M* - *nad2* | 14 | *M* - *nad2* | 14 | *M* - *nad2* | 14 | *M* - *nad2* | 14 | *M*- *nad2* | 14 | *Q*-*M* | 13 |
| *nad2*-*W* |  | *nad2*-*W* |  | *nad2*-*W* |  | *nad2*-*W* |  | *nad2*-*W* |  | *nad2*-*W* |  | *M*-*nad2* | 14 |
| *W*-*Y* | 15 | *W*-*Y* | 15 | *W*-*Y* | 15 | *W*-*Y* | 15 | *W*-*Y* | 15 | *W*-*Y* | 15 | *nad2*-*Y* |  |
| *Y*-*C* | 16 | *Y*-*C* | 16 | *Y*-*C* | 16 | *Y*-*C* | 16 | *Y*-*C* | 16 | *Y*-*C* | 16 | *Y*-*C* | 16 |
| *C*-*cox1* | 17 | *C*- *cox1* | 17 | *C*- *cox1* | 17 | *C*- *cox1* | 17 | *C*- *cox1* | 17 | *C*- *cox1* | 17 | *C*- *cox1* | 17 |

**Table S8. Input file for MLGO analysis using gene order for phylogenetic relationships. within spiders.**

> *Limulus polyphemus*

1 2 3 4 5 6 7 8 9 10 11 12 13 14 -15 -16 -17 -18 -19 20 -21 22 23 24 -25 -26 -27 28 -29 -30 31 32 -33 34 35 36 -37 -38

> *Lyrognathus crotalus*

1 2 3 4 5 6 7 8 9 -26 12 10 13 11 14 -15 -16 -17 -18 -19 -21 22 23 24 20 -25 -27 28 -29 -30 32 -33 31 34 35 36 -38 -37

> *Phyxioschema suthepium*

1 2 3 4 5 6 7 8 9 -26 12 10 13 11 14 -15 -16 -17 -18 -19 -21 22 23 24 20 -25 -27 28 -29 -30 -32 -33 31 34 35 36 -38 -37

> *Calisoga longitarsis*

1 2 3 4 5 6 7 8 9 -26 12 10 13 11 14 -15 -16 -17 -18 -19 -21 22 23 24 20 -25 -27 28 -29 -30 -32 -33 31 34 35 36 -38 -37

>*Ornithoctonus huwena*

1 2 3 4 5 6 7 8 9 -26 12 10 13 11 14 -15 -16 -17 -18 -19 -21 22 23 24 20 -25 -27 28 -29 -30 -32 -33 31 34 35 36 -38 -37

> *Hypochilus thorelli*

1 2 3 4 5 6 7 8 9 -26 12 10 13 11 14 -15 -16 -17 -18 -19 -21 22 23 24 20 -25 -27 28 -29 -30 32 -33 31 34 35 36 -38 -37

> *Pholcus phalangioides*

1 2 3 4 5 6 7 8 9 -26 12 10 13 11 14 -15 -16 -17 -18 -19 -21 22 23 24 20 -25 -27 28 -29 -30 32 -33 31 34 35 36 -38 -37

> *Neoscona theisi*

1 2 3 4 5 6 7 8 9 -26 12 10 13 11 14 -15 -16 -17 -18 -19 -21 22 32 23 24 20 -25 -27 28 -29 -30 -33 31 34 35 36 -38 -37

> *Oxytate striatipes*

1 2 3 4 5 6 7 8 9 -26 12 10 13 11 14 -15 -16 -17 -18 -19 -21 22 32 23 24 20 -25 -27 28 -29 -30 -33 31 34 35 36 -38 -37

> *Araneus angulatus*

1 2 3 4 5 6 7 8 9 -26 12 10 13 11 14 -15 -16 -17 -18 -19 -21 22 32 23 24 20 -25 -27 28 -29 -30 -33 31 34 35 36 -38 -37

> *Selenops bursarius*

1 2 3 4 5 6 7 8 9 26 12 10 13 11 14 -15 -16 -17 -18 -19 -21 22 32 23 24 20 -25 -27 28 -29 -30 -33 31 34 35 36 -38 -37

> *Oxyopes sertatus*

1 2 3 4 5 6 7 8 9 -26 12 10 13 11 14 -15 -16 -17 -18 -19 -21 22 32 23 24 20 -25 -27 28 -29 -30 -33 31 34 35 36 -38 -37

> *Wadicosa fidelis*

1 2 3 4 5 6 7 8 9 -26 12 10 13 11 14 -15 -16 -17 -18 -19 -21 22 32 23 24 20 -25 -27 28 -29 -30 -33 31 34 35 36 -38 -37

> *Argyroneta aquatica*

1 2 3 4 5 6 7 8 9 -26 -12 10 13 11 14 -15 -16 -17 -18 -19 -21 22 32 23 24 20 -25 -27 28 -29 -30 -33 31 34 35 36 -38 -37

> *Agelena silvatica*

1 2 3 4 5 6 7 8 9 13 11 14 -26 10 12 -15 -16 -17 -18 -19 -21 22 -32 23 24 20 -25 -27 28 -29 -30 -33 31 34 35 36 -38 -37

> *Carrhotus xanthogramma*

1 2 3 4 5 6 7 8 9 -26 12 14 10 13 11 -15 -16 -17 -18 -19 36 -21 22 32 23 24 20 -25 -27 28 -29 -30 -33 31 34 35 -38 -37

> *Tetragnatha maxillosa*

1 2 3 4 5 6 7 8 9 -26 12 10 13 11 14 -15 -16 -17 -18 -19 -21 22 32 23 24 20 -25 -27 28 -29 -30 -33 31 34 35 36 -38 -37

> *Liphistius erawan*

1 2 3 4 5 6 7 8 9 10 11 12 13 14 -15 -16 -17 -18 -19 20 -21 22 23 24 -25 -26 -27 28 -29 -30 31 32 -33 34 35 36 -37 -38

**Figure S1. Comparative RSCU analysis of the PCGs of spider mitochondrial genomes.** The figure was edited in Adobe Photoshop CS 8.0.


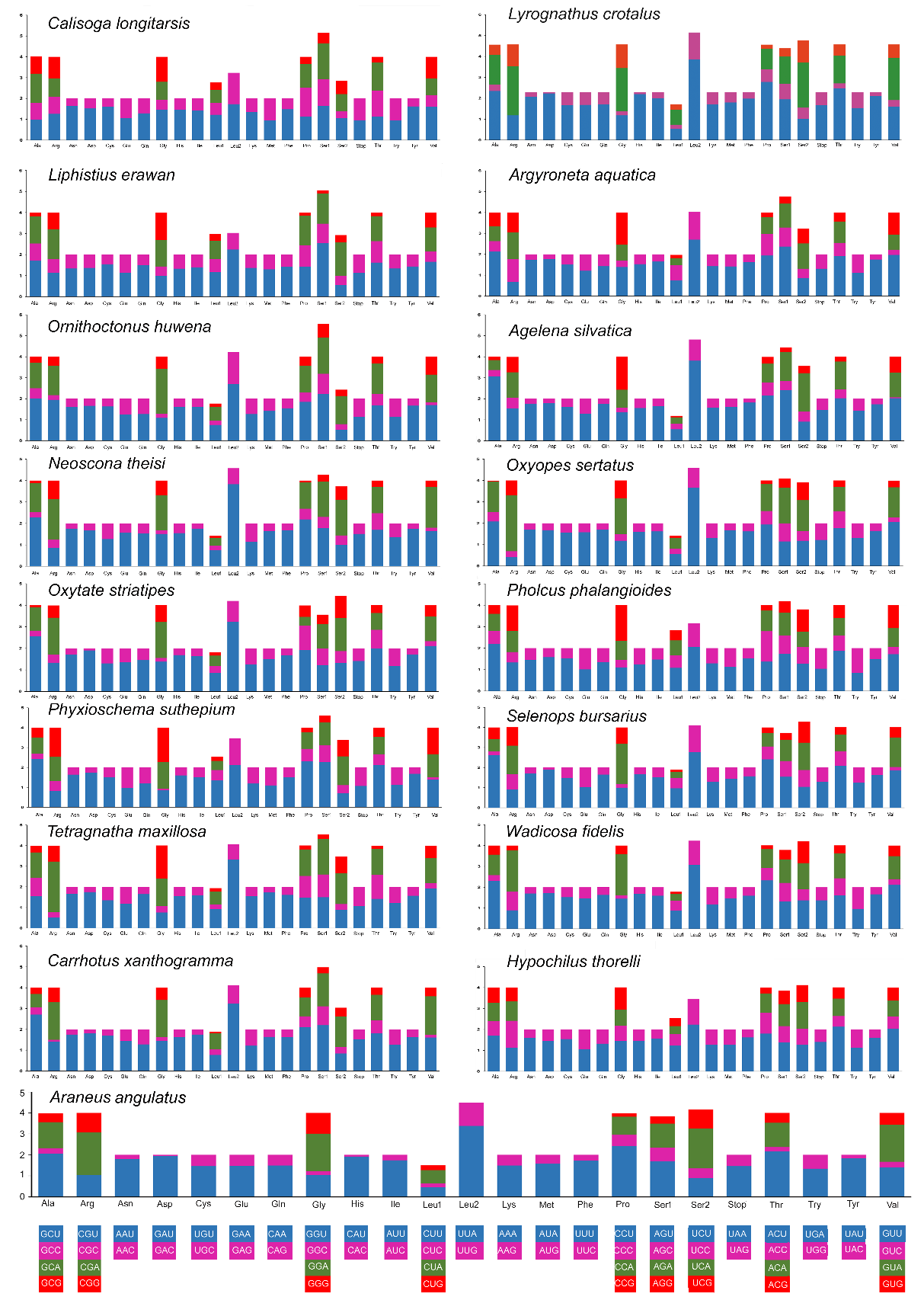
**Figure S2. ENC and GC3 plotting of all PCGs.** The figure was edited in Adobe Photoshop CS 8.0.

**
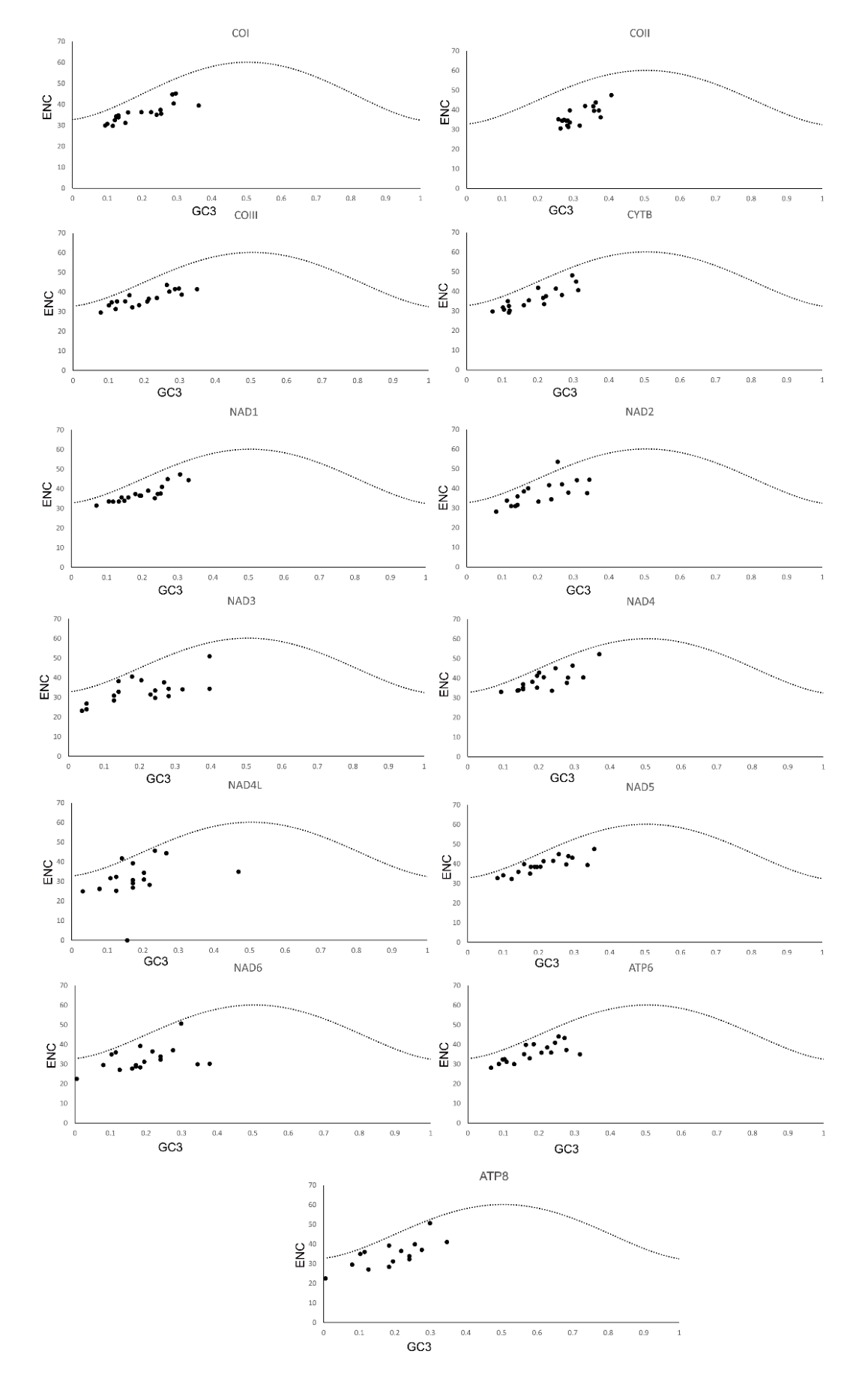
**

**Figure S3. Secondary structures of the 22 tRNA genes of *L. crotalus* mitochondrial genomes.** The tRNAs are represented by full names and IUPAC-IUB single letter amino acid codes. The details of stem and loop is mentioned for one tRNA Tryptophan which is applicable for all tRNAs secondary structures. The figure was edited in Adobe Photoshop CS 8.0.

**
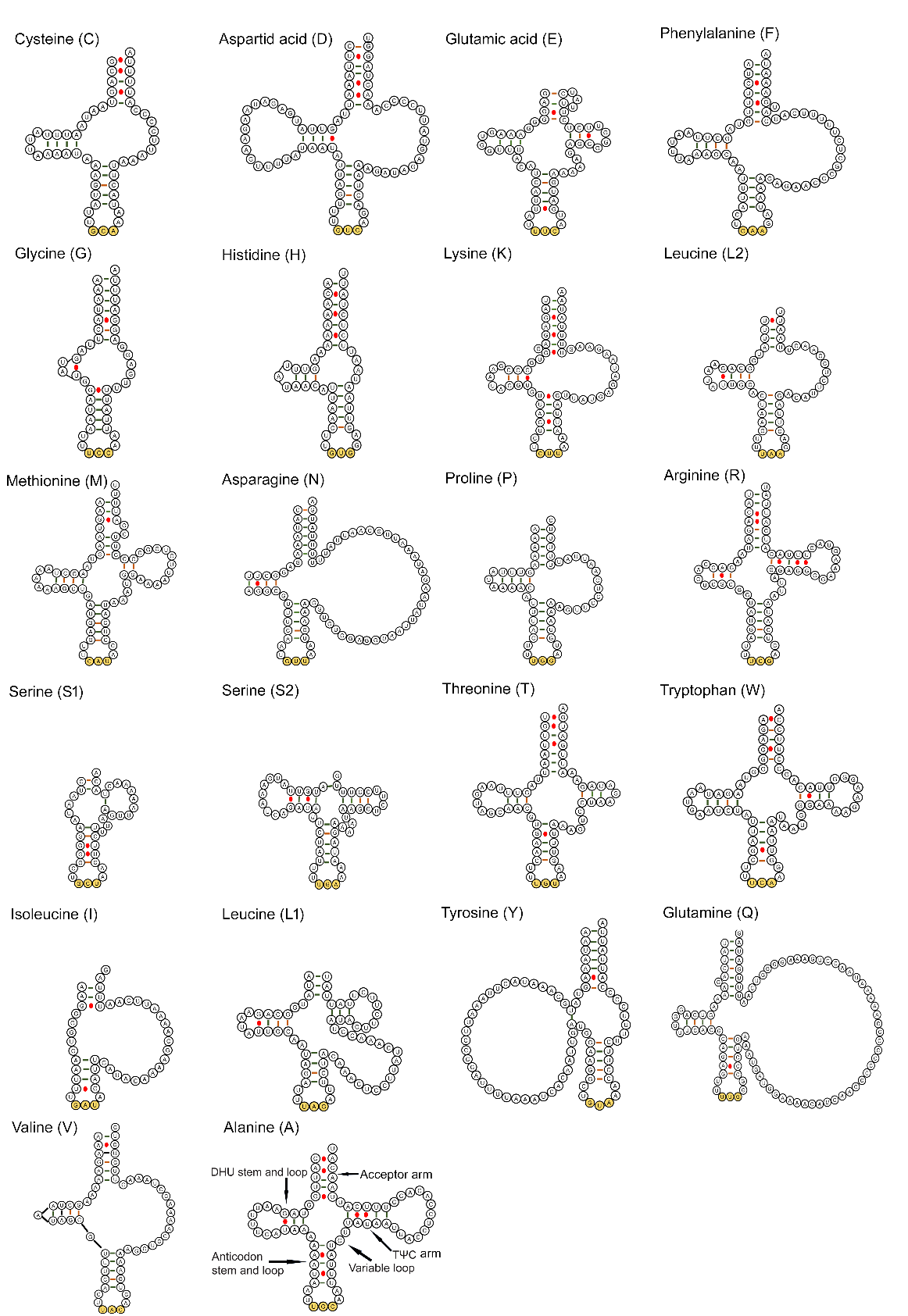
**

**Figure S4. Bayesian Inference inferred by 13 PCGs of spider mitochondrial genomes.** The tree is drawn to scale with posterior probabilities (BI-1/BI-3) indicated along with the branches. The figure was edited in Adobe Photoshop CS 8.0.


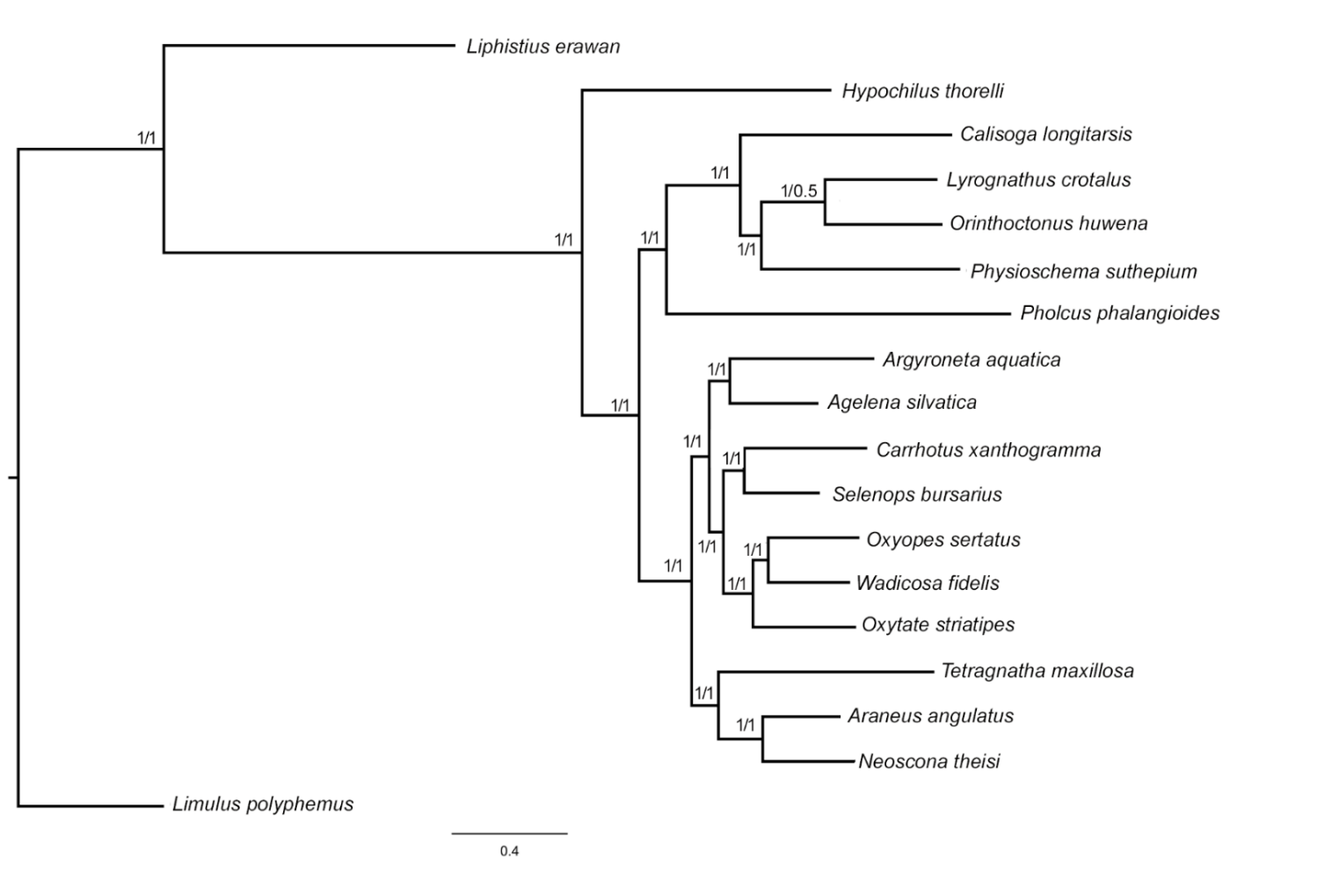


**Figure S5. Maximum likelihood** **tree inferred by 13 PCGs of spider mitochondrial genomes.** The tree is drawn to scale with bootstrap values (ML-1/ML-3) indicated along with the branches. The figure was edited in Adobe Photoshop CS 8.0.


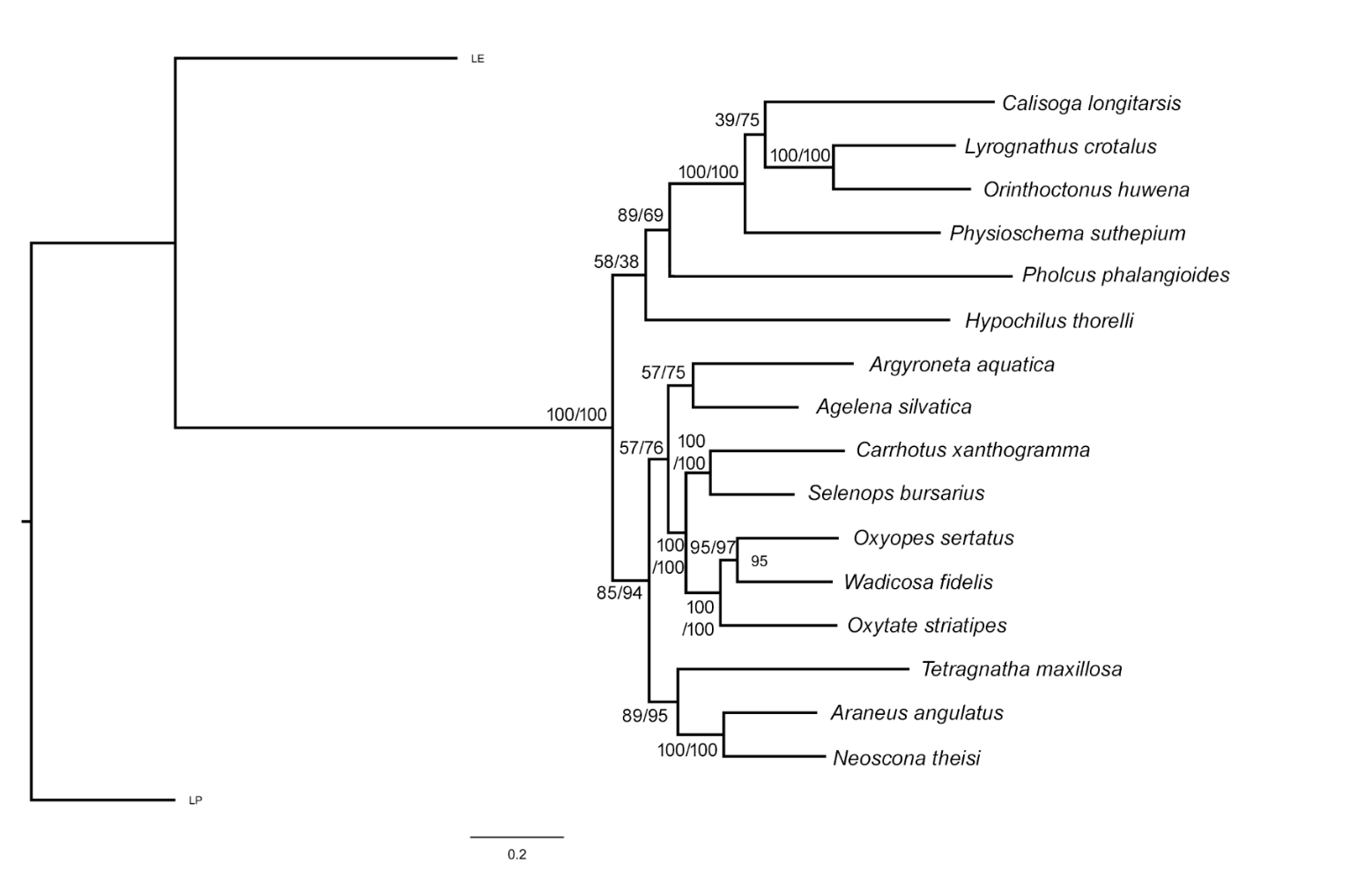


**Figure S6. Maximum likelihood** **tree (ML-2) inferred by 13 PCGs of spider mitochondrial genomes.** The tree is drawn to scale with bootstrap values indicated along with the branches. The figure was edited in Adobe Photoshop CS 8.0.


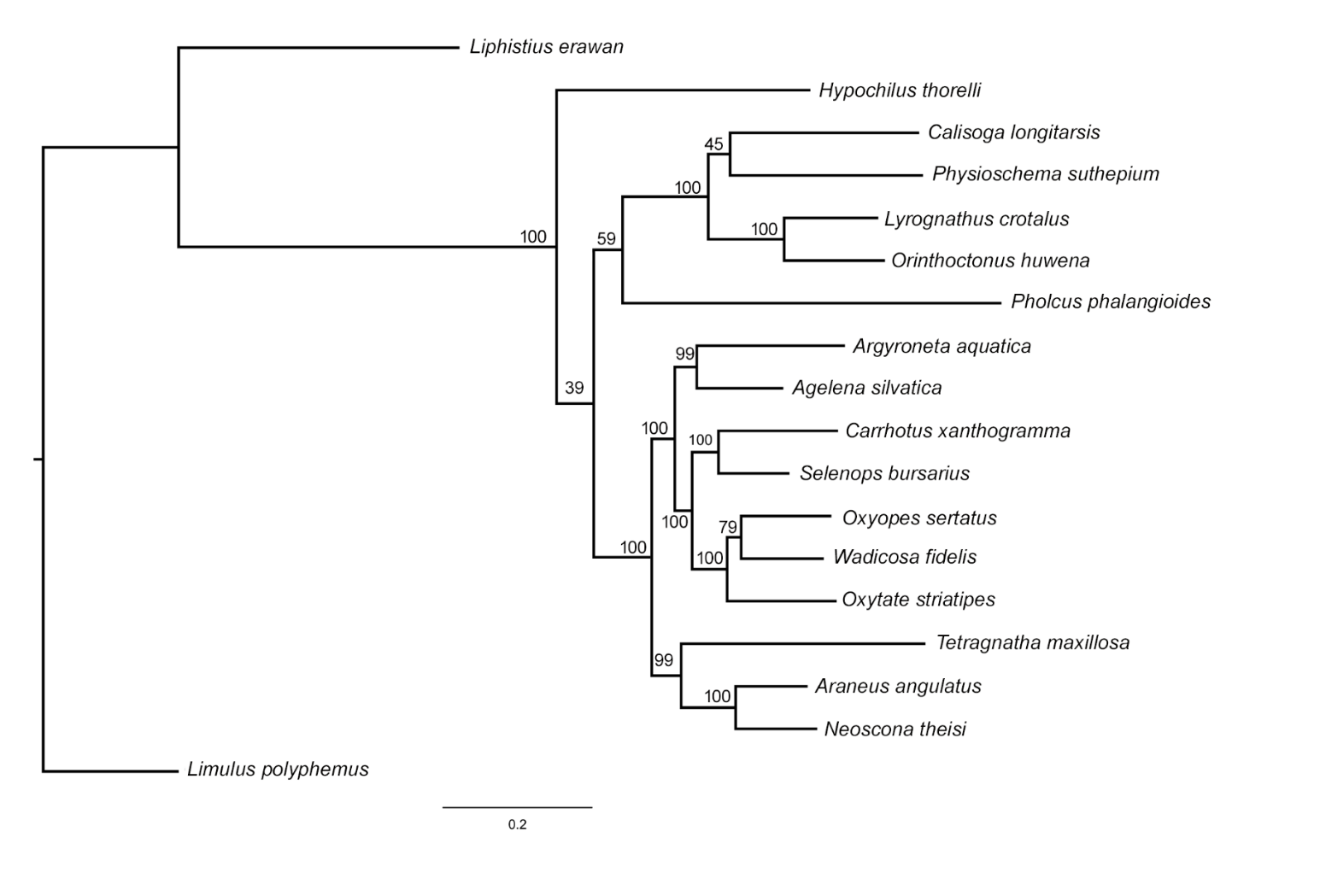

Supplement: Supplementary file 1 — Supplementary Info. [file 41598_2019_57065_MOESM1_ESM.docx]
